# Hepatitis C virus leaves an epigenetic signature post cure of infection by direct-acting antivirals

**DOI:** 10.1371/journal.pgen.1008181

**Published:** 2019-06-19

**Authors:** Shira Perez, Antony Kaspi, Tom Domovitz, Ateret Davidovich, Anat Lavi-Itzkovitz, Tomer Meirson, Jacinta Alison Holmes, Chia-Yen Dai, Chung-Feng Huang, Raymond T. Chung, Assy Nimer, Assam El-Osta, Gur Yaari, Salomon M. Stemmer, Ming-Lung Yu, Izhak Haviv, Meital Gal-Tanamy

**Affiliations:** 1 Molecular Virology Lab, Azrieli Faculty of Medicine in the Galilee, Bar-Ilan University, Safed, Israel; 2 Cancer Personalized Medicine and Diagnostic Genomics Lab, Azrieli Faculty of Medicine in the Galilee, Bar-Ilan University, Safed, Israel; 3 Epigenetics in Human Health and Disease Laboratory, Department of Diabetes, Central Clinical School, Monash University, Melbourne, Australia; 4 Bioengineering, Faculty of Engineering, Bar-Ilan University, Ramat-Gan, Israel; 5 Drug Discovery Laboratory, Azrieli Faculty of Medicine in the Galilee, Bar-Ilan University, Safed, Israel; 6 Liver Center, Division of Gastroenterology, Massachusetts General Hospital, Harvard Medical School, Boston, Massachusetts, United States of America; 7 Hepatobiliary Division, Department of Internal Medicine and Hepatitis Center, Kaohsiung Medical University Hospital, Kaohsiung, Taiwan; 8 School of Medicine and Hepatitis Research Center, College of Medicine, and Center for Cancer Research and Center for Liquid Biopsy, Kaohsiung Medical University, Kaohsiung, Taiwan; 9 Internal Medicine Department A, Western Galilee Medical Center, Naharyia, and Azrieli Faculty of Medicine in the Galilee, Bar-Ilan University, Safed, Israel; 10 Hong Kong Institute of Diabetes and Obesity, Prince of Wales Hospital, The Chinese University of Hong Kong, Hong Kong SAR; 11 Davidoff Center, Rabin Medical Center, Beilinson Campus, Petach Tikva, and Sackler Faculty of Medicine, Tel Aviv University, Tel Aviv, Israel; 12 College of Biological Science and Technology, National Chiao Tung University, Hsin-Chu, Taiwan; 13 Institute of Biomedical Sciences, National Sun Yat-Sen University, Kaohsiung, Taiwan; UCL, UNITED KINGDOM

## Abstract

The increasing worldwide prevalence of Hepatocellular carcinoma (HCC), characterized by resistance to conventional chemotherapy, poor prognosis and eventually mortality, place it as a prime target for new modes of prevention and treatment. Hepatitis C Virus (HCV) is the predominant risk factor for HCC in the US and Europe. Multiple epidemiological studies showed that sustained virological responses (SVR) following treatment with the powerful direct acting antivirals (DAAs), which have replaced interferon-based regimes, do not eliminate tumor development. We aimed to identify an HCV-specific pathogenic mechanism that persists post SVR following DAAs treatment. We demonstrate that HCV infection induces genome-wide epigenetic changes by performing chromatin immunoprecipitation followed by next-generation sequencing (ChIP-seq) for histone post-translational modifications that are epigenetic markers for active and repressed chromatin. The changes in histone modifications correlate with reprogramed host gene expression and alter signaling pathways known to be associated with HCV life cycle and HCC. These epigenetic alterations require the presence of HCV RNA or/and expression of the viral proteins in the cells. Importantly, the epigenetic changes induced following infection persist as an "epigenetic signature" after virus eradication by DAAs treatment, as detected using in vitro HCV infection models. These observations led to the identification of an 8 gene signature that is associated with HCC development and demonstrate persistent epigenetic alterations in HCV infected and post SVR liver biopsy samples. The epigenetic signature was reverted in vitro by drugs that inhibit epigenetic modifying enzyme and by the EGFR inhibitor, Erlotinib. This epigenetic “scarring” of the genome, persisting following HCV eradication, suggest a novel mechanism for the persistent pathogenesis of HCV after its eradication by DAAs. Our study offers new avenues for prevention of the persistent oncogenic effects of chronic hepatitis infections using specific drugs to revert the epigenetic changes to the genome.

## Author summary

Hepatitis C virus (HCV) is a leading cause of hepatocellular carcinoma (HCC) in western countries. While direct acting antivirals (DAAs) therapy for HCV efficiently eradicates the infection, sustained virological response (SVR) following anti-HCV treatment does not eliminate the risk for HCC development. With the 3–4 million newly infected cases each year, it is estimated that HCV-associated disease burden remains high in the next decade and the population of post-DAA-based SVR patients is expected to grow exponentially in the near future. Therefore, Post-SVR HCC is an emerging problem with an urgent unmet need for the elucidation of its molecular mechanisms for therapeutic target and biomarker discovery. We demonstrate that HCV-induce epigenetic missregulation. These HCV-induced epigenetic changes reprogram host gene expression and persist as an "epigenetic signature" following virus eradication. Treatment of HCV-cured cells with specific inhibitors reverted the epigenetic signature. These results suggest a "hit and run" scenario that may explain why some chronic HCV infected patients do proceed to develop HCC after HCV eradication. Our discoveries provide an insight into the outcomes of HCV infection and HCC development, also following SVR, and novel approaches for its prevention.

## Introduction

Hepatocellular Carcinoma (HCC) represents the fifth-most common cancer worldwide and the second cause of cancer death in men, and its incidence has been increasing over the past few decades [1,2]. Its prognosis and survival rates are poor and treatment options are very limited [1]. Currently, liver transplantation is the most effective treatment, and the only drug that prolongs survival by an average of 3 months is sorafenib [3]. HCC is induced by a number of well recognized etiological agents, mainly Hepatitis C virus (HCV) infection in western countries [1]. HCV is a major public health problem with over 71 million people infected worldwide and at risk for developing life-threatening liver diseases [4]. The mechanisms involved in viral persistence leading to HCC are not fully understood.

The recent approval of powerful direct acting antivirals (DAAs) for the treatment of HCV, has made it the only human tumor virus that can be completely eradicated from infected cells. Indeed, the new interferon-free DAAs regimes have yielded rates of sustained virological response (SVR) of over 90%. Studies have demonstrated that SVR following HCV-treatments reduced the risk for HCC by more than 70% (reviewed in [5]). Strikingly, accumulating evidences demonstrate that the DAAs not necessarily prevent progression to HCC, which remains a prevalent complication in SVR populations [6–8]. Very recently, an HCC risk gene signature applicable to all major HCC etiologies was reported. More limited reversal of HCC-related hepatic gene expression was observed in HCV SVR patients who subsequently developed HCC relative to those who did not develop HCC [7,9]. Indirect mechanisms, such cirrhosis, may be important risk factors in development of HCC after virus eradication. Recent studies demonstrated the potential contribution of host immune mechanisms to HCC progression following SVR by DAAs [10–13]. Whether direct virus-mediated "hit and run" mechanisms contribute to the residual risk for HCC following HCV eradication remains elusive [5].

During each stage of the HCV life cycle, its proteins hijack cellular signaling pathways to promote viral propagation [14]. Recent reports suggested that HCV control cell functions also by extensive alterations in expression of genes involved in cellular networks that are essential for the HCV life cycle [15,16]. Still, the maps of host factors involved in HCV propagation and pathogenesis are incomplete, and the mechanisms governing the massive shift in host transcription programs following HCV infection are ill-defined. These complex virus-host interactions suggest that epigenetic changes may occur following HCV infection.

Epigenetics is controlled by the cumulative effect of post translational modifications (PTMs) of histone subunit and base modifications. As epigenetic state is dynamic, it may be navigated by viruses through redirecting histone modifications, resulting in the reprograming of host cell transcription [17]. Whether the presence of the virus in the cell is required for maintaining the altered epigenetic state and the subsequent oncogenic process remains an open question. Since genomes of tumor viruses may be completely lost during the multistep process of tumorigenesis, it has been postulated that stable epigenetic alterations, termed here the "epigenetic signature", may play a role in the mechanism of virus "hit and run" tumorigenesis [18]. In contrast to mutations in cancer, epigenetic alterations are reversible, and therefore are attractive target for the treatment of cancer [19].

Here, we demonstrate that HCV infection modifies the position of histone modifications, thereby inducing an epigenetic signature that persists following curing the viral infection with DAAs; these changes can be reverted by specific drugs. These findings suggest a direct link between HCV and HCC even following SVR, and may provide an opportunity for prevention of HCC progression.

## Results

### HCV infection induces genome-wide repositioning of histone PTMs that correlates with reprogramed host gene expression

Our experimental strategy included evaluating epigenetic changes following HCV infection, their persistence after curing the cells with DAAs and the potential of specific inhibitors to revert the persistent HCV-induced epigenetic signature. These experiments required a cell culture model that: 1) is susceptible for efficient HCV infection for allowing the occurrence of epigenetic changes, 2) can be maintained in culture for at least two months to allow the long experiments of curing the cell from infection, passaging the cells for one month following cure, and treating the cured cells with specific inhibitors for the reversion of the HCV-induced epigenetic signature. Moreover, using in vitro model for HCV infection enables to identify HCV-specific effects on epigenetic marks, and exclude indirect effects that may be related to cirrhosis and immune mediated mechanisms induced in HCV-infected patients. The only cell culture systems that are susceptible to HCV infection are primary human hepatocytes (PHH) and the human Huh7.5 cells line and its derivatives. Since PHH support HCV infection for only short period and can be maintained in culture for only two to three weeks, we used Huh7.5 cells for the extensive profiling the genome-wide epigenetic alterations induced following HCV infection. This cell line support robust HCV infection and have been extensively used in studies exploring the HCV life cycle, virus-host interactions and viral pathogenesis, which led to important landmarks in HCV research [15,20–22]. First, we sought to evaluate the effect of the infection on epigenetic marks in these cells. The Huh7.5 cells were infected with chimeric 1a/2a virus HJ3-5, which replicates and produces infectious viruses. One week following virus infection, approximately 100% of the cells were positive for HCV, and the infection was maintained for one more week to allow for the epigenetic changes to occur. To map the effect of HCV infection on histone PTMs, we performed chromatin immunoprecipitation followed by next generation sequencing (ChIP-seq) of HCV-infected cells compared to non-infected cells. To locate active chromatin, we used antibodies (Abs) binding to histone H3 acetylated on lysine 9 (H3K9Ac) and tri-methylated lysine 4 (H3K4Me3), while silent chromatin was identified using Abs binding histone 3 tri-methylated on lysine 27 (H3K27Me3) and on lysine 9 (H3K9Me3). We have selected these specific modifications following previous report demonstrating that these epigenetic markers determine liver specific epigenetic profile [23].

**Fig 1:**
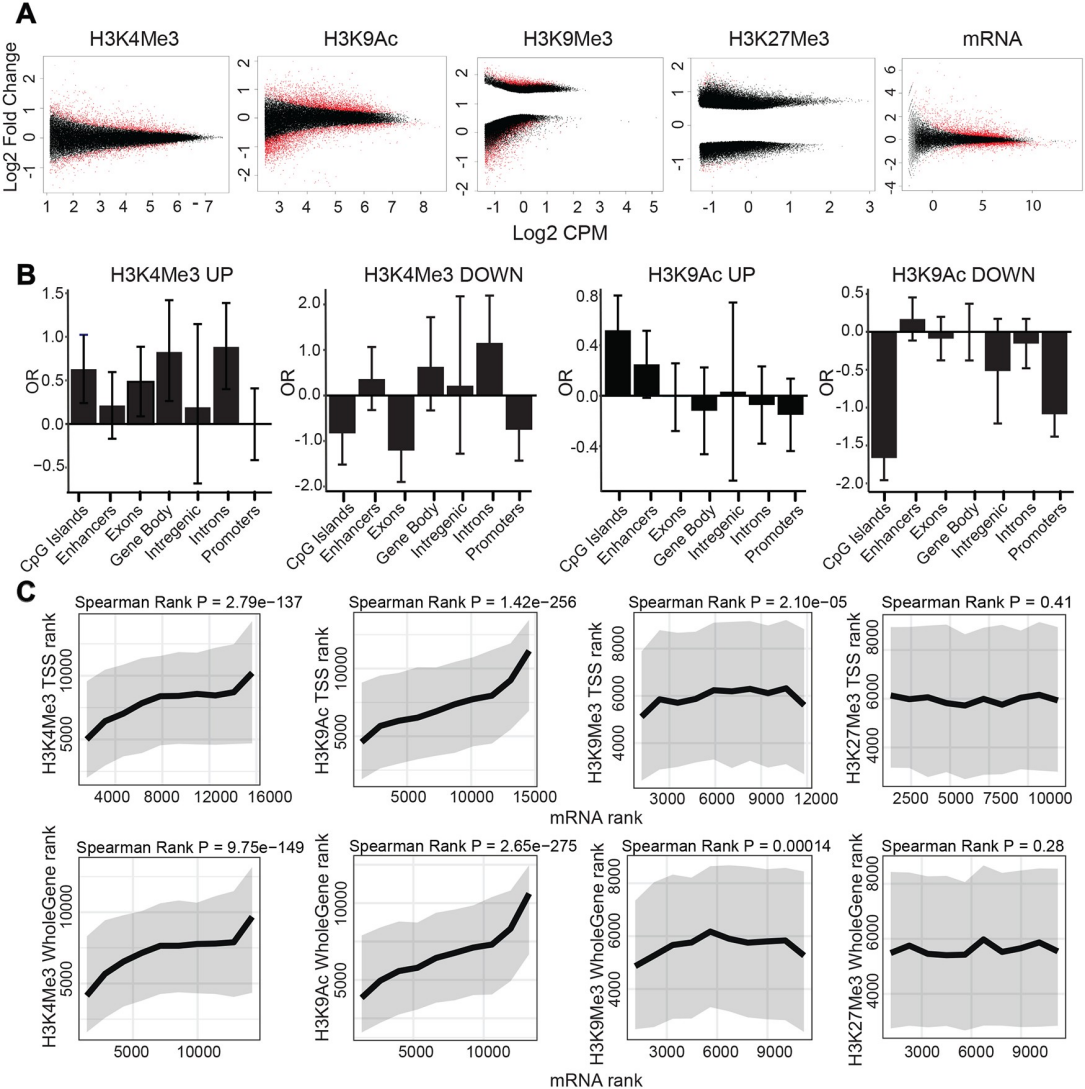
Differential genome-wide distribution of epigenetic markers between HCV infected vs. non-infected cells and its integration with gene expression. (A) Change in genome-wide distribution of epigenetic marks following HCV infection (100% HCV infected Huh7.5 cells, generated as described in the Methods) determined by ChIP-seq experiments, as well as change in gene expression determined by RNA-seq experiment. The plots show the number of counts per million (CPM- the log2 of the read abundance determined by edgeR) for each peak versus the log2 fold change between infected and non-infected cells for each peak. Regions subjected to significant changes are shown in red (adjusted p-value < 0.05), and nonsignificant changes are shown in black. The plots show significant changes in H3K4Me3, H3K9Ac, H3K9Me3 and mRNA expression following HCV infection in Huh7.5 cells. We identified 2,798 kbp of 1221 differential peaks for the epigenetic marker H3K4Me3 (783 up and 438 down), 7,037 kbp of 3827 differential peaks for the epigenetic marker H3K9Ac (1475 up and 2352 down) and 9,028 kbs of 9024 differential peaks for the epigenetic marker H3K9Me3 (2573 up and 6451 down) (p-value < 0.05, Log2FC ≥0.5 and Log2FC ≤ -0.5). Plots demonstrate an average of three to five biological replicates for each ChIP-seq and six biological replicates for RNA-seq experiment. (B) Enrichment of H3K4Me3 and H3K9Ac in genomic features (CpG islands, Enhancers, Exons, gene body, Intergenic, Introns and Promotors). Peaks were compared using a Fisher exact test to a background set which was generated by taking 1000 random non-significant regions (p >0.9). Peaks were defined as p-value < 0.001 for both increased and decreased expression. The Y axis is the log2 Odds Ratio(OR). The error bars indicate 95% confidence intervals. (C) Integration of RNA-seq with H3K4Me3, H3K9Ac, H3K9Me3 and H3K27Me3 ChIP-seq data analysis. Differential mRNA rank was compared to differential ChIP-seq ranks at the TSS or whole gene regions. Ranks were calculated by signed -log10, as determined by edgeR (Ranked from most significant decrease to the most significant increase for: mRNA (x axis) vs ChIP signal (y axis)). Rank order bins of 1000 mRNA contigs each were generated. For each bin, the median (thick line) and interquartile range (grey area) are shown for the matched differential ChIP-Seq ranks. Presented are correlations calculated by Spearman rank. For each ChIP, three to five biological replicates were conducted and for RNA-seq six biological replicates were performed.

The analysis of differential peaks for HCV-infected compared to non-infected cells is shown in Fig 1 demonstrating the number of counts per million (CPM- the log2 of the read abundance determined by edgeR) for each peak versus the log2 fold change between infected and non-infected cells. Significant changes are shown as red dots in Fig 1A. Genome-wide distribution of epigenetic marks shows significant changes in active chromatin markers H3K4Me3 and H3K9Ac and the silent chromatin marker H3K9Me3 following HCV infection. We did not observe significant changes in the silent chromatin epigenetic marker H3K27Me3 (Fig 1A). Intersection of these data with annotated genomic features revealed that the H3K4Me3 regions are enriched at gene body, including introns and exons, while H3K9Ac regions are mainly enriched at regulatory elements, including CpG islands and enhancers (Fig 1B). This annotation defined the differentially modified genes (DMGs; genes in the vicinity of differential ChIP-seq peaks) induced by HCV infection for each ChIP-seq experiment (as described in the Methods). Interestingly, for both H3K9Ac H3K4Me3 regions we did not find significant enrichment in intergenic regions, suggesting that the epigenetic changes induced by HCV are not randomly distributed throughout the genome, and are mainly directed to regions containing genes and regulatory elements.

To evaluate the correlation between the changes in epigenetic profile and the changes in gene expression following HCV infection, we first analyzed the transcriptome by RNA-seq comparing HCV-infected to non-infected cells. The RNA-seq analysis of differential peaks (HCV-infected compared to non-infected cells) identified 2672 differentially expressed genes (DEGs) (using a threshold of p < 0.05, Log2FC ≥1.5 and Log2FC ≤ -1.5) (Fig 1A, in red). Of these, HCV infection induced upregulation of 1865 genes (Fig 1A, red dots, Log2 Fold Change>1.5) and downregulation of 807 genes (Fig 1A, red dots, Log2 Fold Change<-1.5). We have generated a ranked list of DMGs (for ChIP-seq signal at a specific gene) and DEGs (for mRNA) (Ranked from most significant decrease to the most significant increase). Plotting the differential mRNA rank (rank position of the DEG following HCV infection), against the differential ChIP-seq rank (rank position of the DMG following HCV infection), demonstrated positive correlation between H3K4Me3 and H3K9Ac -enriched DMGs and the mRNA score at TSS regions (Fig 1C, Spearman rank: p = 2.9e-137 and p = 1.42e-256, respectively) as well as in whole gene regions (Fig 1C, Spearman rank: p = 9.75e-149 and p = 2.65e-275, respectively). We observed a modest negative correlation at the high rank of H3K9Me3-enriched regions and mRNA score at TSS regions (Fig 1C, Spearman rank: p = 2.10e-05) as well as in whole gene regions (Fig 1C, Spearman rank: p = 0.00014). No correlation was observed between mRNA score and the transcriptional repression marker H3K27Me3 at TSS regions (Fig 1C, Spearman rank: p = 0.41) as well as in whole gene regions (Fig 1C, Spearman rank: p = 0.28). These results show that level of change in expression of genes following HCV infection is correlated with the level of change in epigenetic marker on the chromatin following HCV infection at the position of these same genes. Intersection between H3K4Me3, H3K9Ac regions and mRNA regions in whole gene or TSS regions demonstrated overlap between H3K9Ac and H3K4Me3-DMGs and upregulated DEGs, while there was no overlap with down regulated genes (S1 Fig). These data are consistent with the known role of these epigenetic markers in the establishment of open chromatin and transcription activation. Collectively, these results confirm the correlation between the genome-wide change in distribution of PTMs H3K9Ac and H3K4Me3 and DEGs following HCV infection.

To validate the DMGs based on the H3K9Ac and H3K4Me3 ChIP-seq data, and DEGs, we selected a list of DMGs changes in H3K9Ac ChIP-seq data (listed in S2A Fig), or H3K4Me3 ChIP-seq data (listed in S2B Fig), or both; in total 21 DMGs that surfaced as they were the top significantly altered DEGs following HCV infection (p < 1.81E-06, Log2FC ≥1 or Log2FC ≤ -1). These genes are implicated in cell differentiation, motility, growth, proliferation, cell death, metabolism, liver development, stress, cell transformation and immune responses (S1 and S2 Tables). For validation of DMGs we performed ChIP-qPCR with primers targeting the peak of each gene according to the ChIP-seq data, and for validation of DEGs, we performed RT-PCR with gene specific primers. Overall, the qPCR gene-specific method validated the upregulation or down regulation signals we observed in the genome-wide analysis (Fisher exact, p = 0.00001). In addition, increased or decreased levels of open chromatin markers H3K9Ac and H3K4Me3 were aligned with increase or decrease in gene expression, respectively (Fisher exact, p = 0.00001) (S2 Fig). As control, we selected a list of 5 genes that their mRNA levels were not changed following infection by qPCR analysis (S2D Fig).

We also validated the effect of HCV on gene expression in primary human hepatocytes (PHH) that were infected with HCV for one week, until most of the cells (60–70%) were positive for HCV infection by immunostaining (S3A Fig) and HCV RNA replication was detected (S3B Fig). We observed correlation between the changes in expression of panel of genes in Huh7.5 cells compared to PHH following HCV infection (Fisher exact, p = 0.0002) (S3C Fig). To evaluate the effect of longer period of HCV infection (compared to 2 weeks in Huh7.5 cells) on epigenetic alteration, we evaluated the DMGs and DEGs in hepatocyte-like cells generated by maintaining Huh7.5 cells in human serum (Huh7.5-HS). These cells were previously reported to differentiate into hepatocyte-like cells and produce high titers of infectious HCV. The infection in these cells was reported to be stable for at least 65 days [24] as also demonstrated here in S4A Fig. Importantly, gene expression profiles of these hepatocyte-like cells significantly overlap with those of primary human hepatocytes. Moreover, the transcriptome of these hepatocyte-like cells infected with HCV significantly correlated with a high HCC risk profile among 216 HCV-infected patients [25]. Therefore, these cells were suggested to serve as a convenient model for the study of liver diseases [24,25]. As was previously reported, after culturing the Huh7.5 cells for two weeks with human serum we observed features of cultured primary hepatocytes. The morphology of the cells dramatically changed (more rounded, granulated and bigger) and the level of Albumin expression was increased. Long term infection of 60 days in Huh7.5 cells demonstrated comparable levels of DMGs and DEGs, validating the change in epigenetic profile under conditions that simulate chronic HCV infection (Fisher exact, p<0.01) (S4B and S4C Fig).

HCV is classified into 7 genotypes, with varied distribution throughout the world. The evaluation of epigenetic and gene expression changes were performed using the HJ3-5 virus, a genotypes 1a/2a chimera, since it represent the most prevalent genotype in the US and Europe. An interesting question is whether other HCV genotypes induce similar changes following infection. We used chimeric viruses contain core-NS2 proteins from genotypes 2–7 to infect Huh7.5 cells, and when approximately 100% of the cells were positive for HCV, we performed RT-PCR to evaluate HCV RNA replication and expression of specific genes. Genotypes 2–7 replicated efficiently in Huh7.5, although at lower level than genotype 1 chimeric virus HJ3-5 (S5A Fig). Gene expression profiles following infection with genotypes 2–7 were generally similar to the gene expression profile of genotype 1a infection, except for genotype 2 that also replicated at lower efficiency (S5B Fig).

### HCV infection induces an epigenetic signature that is not reverted after curing the infection with DAAs

**Fig 2:**
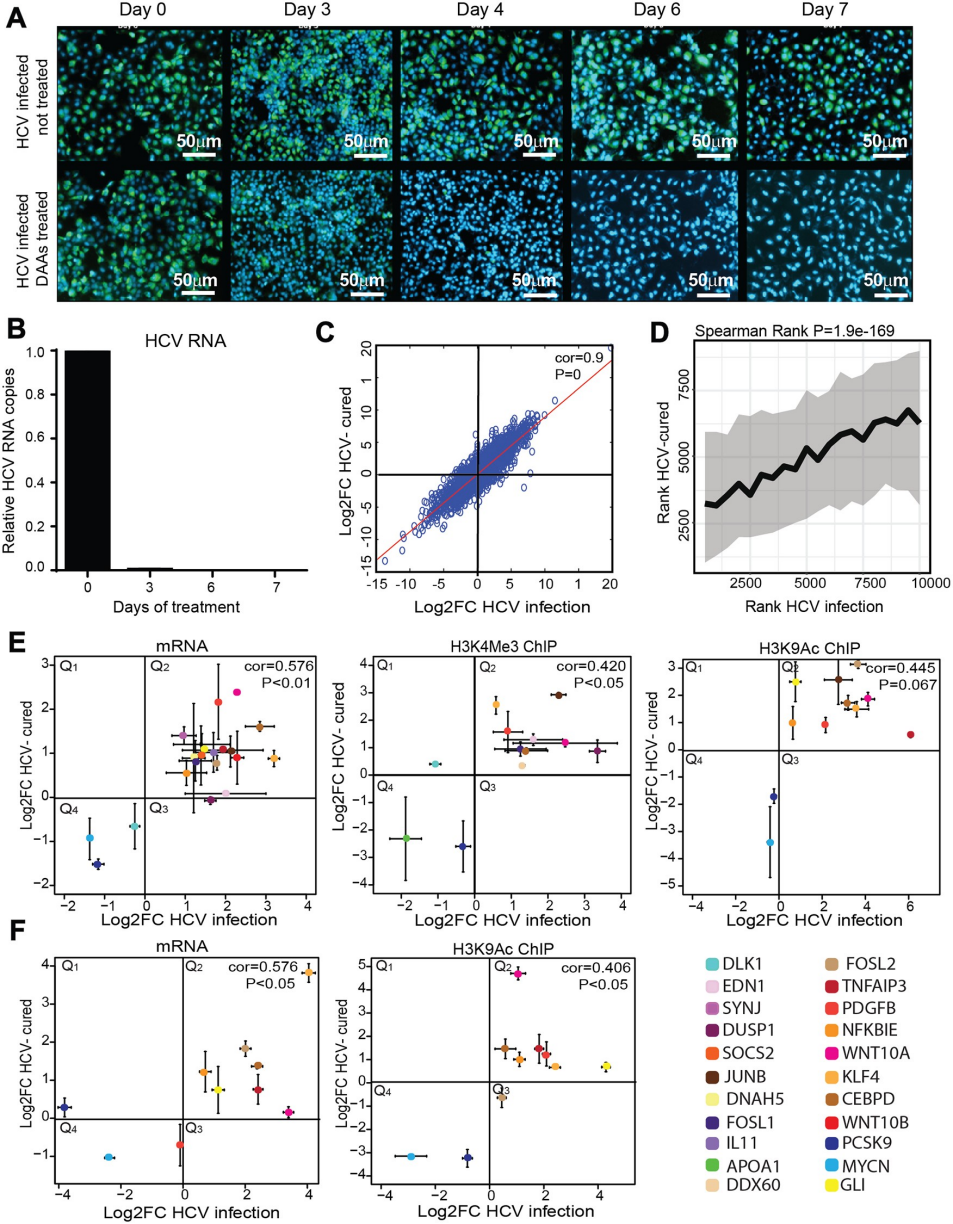
Epigenetic signature following cure of HCV by DAAs. (A) Immunostaining to evaluate therapeutic potential of DAAs in HCV-infected cells. HCV-infected Huh7.5 cells, treated or not treated with DAAs were stained with HCV-positive serum and anti-human 488 Alexa fluor as secondary antibody to follow the eradication of HCV infection following treatment. Infection was visualized by fluorescence microscopy. Scale bars: 50μm. (B) The therapeutic potential of DAAs in HCV infected cells was evaluated by qRT-PCR. Levels of HCV RNA in the cells were quantified by qRT-PCR using primers for the HCV RNA 3’ UTR. Relative HCV RNA copies are calculated for Huh7.5 cured cells compared to non-infected Huh7.5 cells (also treated with DAAs) per ng of total cellular RNA. Differential expression was calculated using the equation of 2^(-ΔΔCt)^, with the GAPDH as an endogenous control. Means mRNA levels of HCV are shown ± SD from three independent experiments. (C) Scatter plot of RNA level in HCV-cured cells (fold change was calculated compared to control cells) compared to HCV-infected cells (fold change was calculated compared to non-infected cells) and evaluated by qRT-PCR (Spearman Rank cor = 0.91, p = 0). Four biological replicates were performed. (D) Correlation between differential peaks in ChIP-seq of H3K9Ac in HCV-cured cells (compared to control cells) and HCV-infected cells (compared to non-infected cells). Differential regions were identified using edgeR. Each of the experiments was filtered for the most significant 60000 loci by P value. These were matched between the two experiments leaving 10000 loci. These results were then ranked by the directional -log10(PValue), for each experiment. The X rank was broken into 20 consecutive bins. For each of these bins the median and interquartile range of the matched ranks in Y were calculated and plotted (Spearman rank: p = 1.9e-169). Plots demonstrate an average of three to five biological replicates for each ChIP-seq. (E) Scatter plot of RNA level for specific genes in Huh7.5-cured cells (fold change was calculated compared to DAAs treated control cells) compared to HCV-infected cells (fold change was calculated compared to non-infected cells) and evaluated by qRT-PCR. Differential expression was calculated using the equation of 2^(-ΔΔCt)^, with the GAPDH as an endogenous control. ChIP for H3K4Me3 and H3K9Ac histone modifications were performed in HCV-cured cells (fold change was calculated compared to DAAs treated control cells) compared to HCV infected cells (fold change was calculated compared to non-infected cells). The IP DNA level was quantified by qPCR with primers for specific genes. Values were normalized relative to qPCR for these genes following ChIP with normal Rabbit IgG Ab as control. Values are presented in scatter plots representing average ± SD from three biological replicates. Correlations calculated by Spearman rank and p-values are presented. (F) Scatter plot of RNA and H3K9Ac histone modification levels in specific genes in cured Huh7.5-HS cells compared to HCV infected Huh7.5-HS cells performed as described above. Values are presented in scatter plots representing average ± SD from Three biological replicates. Correlations calculated by Spearman rank and p-values are presented.

Since post-translational histone modifications were suggested to contribute to epigenetic memory [26], we suggested that a transient HCV infection may leave an "epigenetic signature" on the host chromatin that is not fully reversed following virus eradication after treatment with DAAs. To explore the reversibility of HCV-induced changes in position of histone PTMs following HCV eradication we treated HCV-infected and non-infected Huh7.5 cells with the all oral interferon-free FDA approved anti-HCV DAAs combination containing: dasabuvir, ombitasvir, paritaprevir and ritonavir, which achieves SVR rates greater than 95% in HCV genotype 1– infected patients [27,28]. HCV-infected cells were treated with an effective non-cytotoxic concentration (marked in yellow in S6 Fig) for 7 days; virus eradication was achieved within 3–4 days of treatment as demonstrated by no expression of HCV proteins (Fig 2A), or no HCV RNA in treated cells (Fig 2B). Cultures were maintained for 1 month after HCV eradication (termed here as "cured cells") before analysis. First, we evaluated the effect of HCV eradication on gene expression. We performed RNA-seq on HCV-cured cells, and compared their gene expression profile to DAAs-treated non-infected cells as control. Plotting the Log2 fold change of the list of genes changes following HCV infection (presented in Fig 1) against the Log2 fold change of the list of genes changes in DAAs-treated cured cells show a highly significant correlation in mRNA levels (Spearman rank, cor = 0.91 p = 0) (Fig 2C). We also performed ChIP-seq experiment to evaluate the level of H3K9Ac levels in DAAs treated cured cells compared to control non-infected cells. Of the 3827 differential peaks identified for the HCV-infected experiment, 776 regions overlapped with the DAAs-cured experiment; most of these (599) are in genic regions. We have generated a ranked list of differential regions (ranked from most significant decrease to the most significant increase) for the HCV-infected experiment (presented in Fig 1) and DAAs-cured experiment. Plotting the two lists demonstrated strong positive correlation between scores of H3K9Ac -enriched regions in HCV-infected and cured cells (Fig 2D, Spearman rank: p = 1.9e-169). Analyzing the overlap between differential genes (P < 0.05, absolute logFC < 0.5, with changes within genes and up to 3kb of the TSS) demonstrated that of the 1322 annotated genes that were changed in the HCV-infection experiment, 752 were also persistently altered in the HCV-cured experiment, providing an estimation of more than 50% of genome-wide HCV-induced epigenetic alterations in DMGs remain as an epigenetic signature.

We also validated specific genes for the RNA-seq and ChIP-seq data. Analysis of mRNA levels for the specific genes (selected as described above) by qPCR demonstrated that for the genes DUSP1 and EDN1 the changes in mRNA levels following infection were reverted to their preinfection levels following cure. However, for the remaining 18 tested genes, the virally induced changes remain persistent in cured cells, demonstrating positive correlation between HCV infected and cured cells (cor = 0.576, p<0.01) (Fig 2E). Analysis of ChIP data for epigenetic markers H3K4Me3 and H3K9Ac followed by qPCR for specific genes in HCV-cured cells, compared to HCV-infected cells, also demonstrated that the alteration induced by infection did not revert in cured cells (cor = 0.42, p<0.5 and cor = 0.445, p = 0.067, respectively). As shown in the scatter plots in Fig 2E, the HCV-induced increased levels of epigenetic marker in DMGs remained high (Q2), and the HCV-induced decreased levels of epigenetic marker in DMGs remained low (Q4) in HCV-cured cells.

We also evaluated the HCV-induced gene expression and epigenetic signature in Huh7.5-HS hepatocytes-like cells. Comparable results were obtained, demonstrating that gene expression levels, as well as H3K9Ac levels, did not revert, or only partially reverted in HCV-cured Huh7.5-HS cells following DAAs treatment (cor = 0.576, p<0.5 and cor = 0.406, p<0.5, respectively) (Fig 2F). These results validate the HCV infection-induced epigenetic signature in the hepatocytes-like cells that exhibit gene expression profile that is close to normal non-neoplastic hepatocytes.

The DAAs replaced the previously-used Interferon-based regimes in treatment of HCV infection. Therefore, we sought to evaluate whether curing HCV infection with Interferon would also maintain the gene expression and epigenetic signature. HCV infected Huh7.5 cells were treated with interferon for three weeks, until no viral RNA or proteins were detected in the cells and evaluated mRNA levels for specific genes in interferon-cured cells compared to interferon treated control cells. Interferon treatment resulted in more efficient reversion, with complete reversion in some genes (S7A Fig). This reversion was also observed in H3K9Ac levels following ChIP and qPCR for specific genes in Interferon-cured cells (S7B Fig).

### HCV-induced epigenetic alterations misregulate host signaling pathways implicated in HCV life cycle and HCC

**Fig 3:**
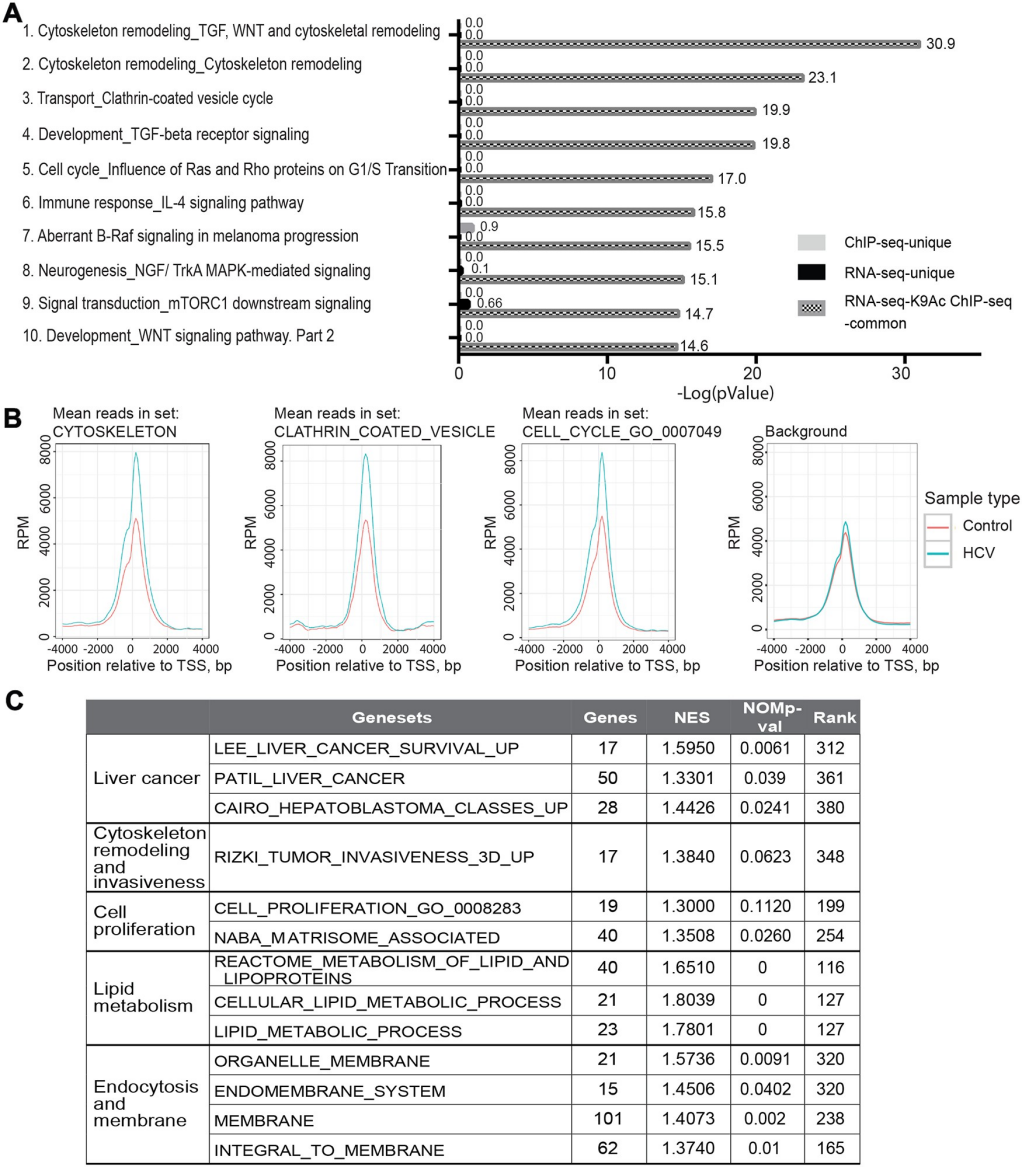
Pathway analysis generated from RNA-seq and H3K9Ac ChIP-seq integration data. (A) Pathway analysis was generated by using both the RNA-seq and H3K9Ac ChIP-seq data. Enrichment analysis consisted of matching gene IDs of possible targets for the "common”(common to the RNA-seq and H3K9Ac ChIP-seq data) and "unique" (unique to the RNA-seq or H3K9Ac ChIP-seq data) sets, with gene IDs in functional ontologies in MetaCore. The probability of a random intersection between a set of IDs the size of the target list with ontology entities is estimated by the p-value of the hypergeometric intersection. Data were generated using the Thompson Reuter MetaCore analysis GeneGo. The most significant pathway maps that are common to both the RNA-seq and H3K9Ac ChIP-seq data are shown. The Log10 of the p-value is presented. (B) Profiles of the combined H3K9Ac Chip-seq data across the TSS regions of genes belonging to the indicated GO gene sets, and a control background generated by ranking all genes by read depth and randomly selecting control genes within 20 rank positions of each gene in the primary gene sets. Each line represents a triplicate. RPM, reads per million. (C) GSEA generated from H3K9Ac ChIP-seq data. A ranked gene list was generated for the differential H3K9Ac ChIP-seq data according to the p value. This ranked list was used for Gene Set Enrichment Analysis (http://software.broadinstitute.org/gsea/index.jsp).

To investigate the signaling pathways that are epigenetically misregulated following HCV infection, we first subjected the outlier RNA-Seq DEG, and H3K9Ac ChIP-seq DMG data for gene ontology (GO) analysis using the MetaCore database from Thomson Reuters (ver. 6.11, build 41105, GeneGo, Thomson Reuters, New York, NY, USA) [29] pathway analysis tool. This tool was selected since it enables to perform gene network enrichment analysis from integration of RNA-seq and H3K9Ac ChIP-seq data. Using the RNA-Seq and H3K9Ac ChIP-Seq data we generated ranked lists of differentially modified genes (DMGs, for ChIP-seq signal at a vicinity of a specific gene; for peaks within gene body, both exons or introns, and within TSS, up to 5kb upstream of the TSS) and differentially expressed genes (DEGs, for RNA-seq signal) (Ranked from most significant decrease to the most significant increase). The two gene lists were uploaded to the MetaCore database, that provided an integrated list of pathways changed both in epigenetic marker and transcription. The full description of the identified pathways, their statistical significance, and the list of genes involved in each pathway that are altered are provided in S3 Table. The results in Fig 3A show the ten most significant pathway maps (ranked by p-value) enriched in both HCV-infection-stimulated DEGs and H3K9Ac ChIP-seq DMGs. The most significant pathways include two cytoskeleton remodeling pathways named: "Cytoskeleton remodeling_TGF, WNT and cytoskeletal remodeling" (-log(p-value)>30) and "Cytoskeleton remodeling" (-log(p-value)>20) (Fig 3A). The third most significant pathway is "Transport_clathrin coated vesicle cycle" (-log(p-value) = 20) (Fig 3A). The other pathways are associated with cell cycle, development, immune response, and other cancer- related pathways (B-Raf, NGF, mTOR/MAPK and WNT signaling) (Fig 3A). Overall, 99% of H3K9Ac DMGs are common with DEGs detected by RNA-seq (FDR<1.96e-13) (Fig 3A and S3 Table). In contrast, the enrichment of genes in these pathways at altered sites unique to the H3K9Ac ChIP-seq or RNA-seq data was not significant (FDR >0.14) (Fig 3A and S1 Table). Intersecting the H3K9Ac Chip-seq DMGs across the TSS regions of genes belonging to the top identified GO genesets cytoskeleton, clathrin mediated endocytosis, and cell cycle, reveal a significantly higher number of peaks in HCV- infected compared to non-infected cells (Fig 3B). No enrichment of peaks was observed in control background analyzing the TSS regions of control set generated by ranking all genes by read depth and randomly selecting control genes within 20 rank positions of each gene in the primary gene sets (Fig 3B). To further investigate the signaling pathways that are misregulated by HCV infection, we next performed Gene Sets Enrichment Analysis (GSEA), which assesses the effect of HCV infection across the complete genome-wide rank (from the largest increase to the largest decrease) on H3K9Ac ChIP-seq DMGs. The genesets with the most significant enrichment along the HCV-infection-stimulated rank are involved in liver cancer, cytoskeleton remodeling and invasiveness, cell proliferation, lipid metabolism, endocytosis and membrane trafficking and epigenetic modifiers (Fig 3C and S8 Fig). These genesets overlap with pathways identified by MetaCore analysis, further validating our findings that HCV infection alters these pathways through epigenetic regulation.

Altogether, these analyses suggest that altered transcription programs induced following HCV infection are regulated by altering histone PTM sites that affect key cellular pathways. These pathways are associated with the HCV lifecycle and HCC, as elaborated in the Discussion.

### The epigenetic signature induced by HCV infection is associated with increased risk for HCC development

**Fig 4:**
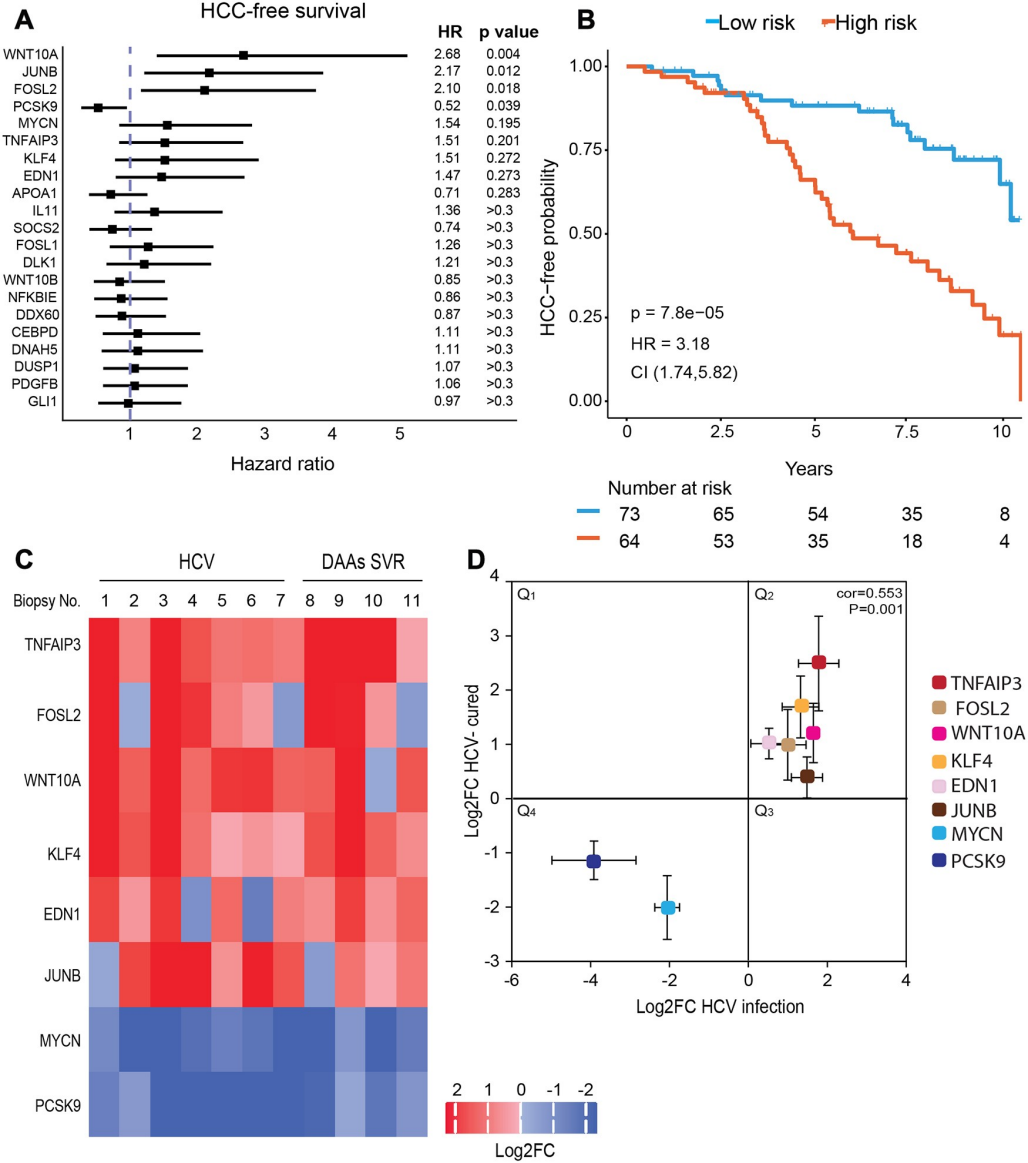
Epigenetic signature in human liver biopsies following cure of HCV infection. (A) Forest plot of hazard ratios (HR) for HCC development among hepatitis C-related early stage cirrhosis samples [30], stratified according to the median gene expression levels. P-values were calculated using log-rank test. Genes are ordered from lowest to highest p-values. The HR, 95% confidence intervals (CI) and p-values are shown. (B) HCC-free survival according to the level of expression of the 8-gene signature (*WNT10A*, *JUNB*, *FOSL2*, *MYCN*, *TNFAIP3*, *KLF4*, *EDN1*, *PCSK9*). P-values were calculated using log-rank test. The HR and 95% CI are shown. (C and D) H3K9Ac ChIP was performed on liver biopsies (clinical data is described in S4 Table). The level of H3K9Ac for specific genes was quantified by qPCR, and values were normalized to ChIP with normal Rabbit IgG Ab only. These levels were quantified in 7 HCV-infected biopsies (No. 1–7) and 4 SVR biopsies (No. 8–11) and compared to the average level of the 6 control biopsies (No. 12–17). (C) Values of Log2FC for H3K9Ac ChIP-qPCR for each sample and each gene versus control are presented as a heatmap. (D) A scatter plot showing for each gene the Log2FC average values for H3K9Ac ChIP-qPCR for the HCV-infected biopsies (No. 1–7) versus the Log2FC H3K9Ac ChIP-qPCR average values for the SVR biopsies (No. 8–11). Correlation was calculated by Spearman rank (cor = 0.553, p = 0.001).

We next aimed to validate these findings in human samples, and evaluate their implications for HCC development. We collected and analyzed 17 liver samples that include: normal liver samples as controls (n = 6), HCV infected liver samples (n = 7) and liver samples from SVR patients with previous HCV infection following DAAs treatment (n = 4). The clinical data for these samples is provided in S4 Table. First, we evaluated the association between expression of the panel of genes we validated to be persistently altered in HCV cured cells (Fig 2), to the development of HCC. We analyzed microarray-based genome-wide gene expression profiles data obtained from liver biopsies from 216 patients with hepatitis C-related Child-Pugh class A cirrhosis [30]. The forest plot in Fig 4A demonstrates that expression of most of these genes correlated with hazard ratio HR>1 that is associated with increased risk for HCC development. The top genes, which demonstrated the lowest p-value and most significant HR, were selected as gene signature that may predict the risk for HCC development pre and post SVR. This gene signature includes *WNT10A*, *JUNB*, *FOSL2*, *MYCN*, *TNFAIP3*, *KLF4* and *EDN1* with HR>1 (high expression correlates with HCC development) and *PCSK9* with HR<1 (low expression correlates with HCC development). We also evaluated the HCC-free survival based on this eight-gene expression signature and stratified the patients into low and high risk groups. The results demonstrate a dramatic significant correlation between acceleration in HCC development in HCV infected patients and altered level of expression of the gene signature, with HR = 3.18 and p = 7.8–05 (Fig 4B). Interestingly, the altered gene expression is associated with increased risk after 2.5 years, implying that this HCV-induced mechanism slowly increases the risk for HCC.

To validate the persistence of the epigenetic changes in this eight genes signature following SVR in patients, we evaluated the alterations in H3K9Ac levels for these genes in liver biopsies described above by performing ChIP-qPCR as described above. The heatmap in Fig 4C present the log2 fold change values for the 8 signature gens for each sample, calculated compared to the average value of the 6 control samples. This heatmap demonstrate that the H3K9Ac level at the genome position of these genes is altered in the HCV-infected samples, compared to average values of the controls (Fig 4C). The level of the epigenetic marker at the position of genes *WNT10A*, *JUNB*, *FOSL2*, *TNFAIP3*, *KLF4* and *EDN1* was increased, and decreased at the position of genes *MYCN* and *PCSK9* (Fig 4C), as was demonstrated by using the in vitro HCV-infection systems (Fig 2E and 2F). Most importantly, these alterations remained persistent in the DAAs treated patients (Fig 4C), also consistent with our in-vitro results. The scatter plot in Fig 4D summarize the result (shown in Fig 4C) for each gene, presenting the Log2FC average values for H3K9Ac ChIP-qPCR for the HCV-infected biopsies (No. 1–7) versus the Log2FC H3K9Ac ChIP-qPCR average values for the SVR biopsies (No. 8–11). The data demonstrates positive correlation between the epigenetic alterations in HCV infected livers and post SVR (Spearman rho = 0.55, p = 0.001). Together, these observations validate the persistent epigenetic and gene expression signatures following SVR by DAAs in clinical samples, and demonstrate its relevance for HCC development.

### The HCV- induced epigenetic alterations require the presence of HCV RNA and/or expression of HCV proteins in the cells

**Fig 5:**
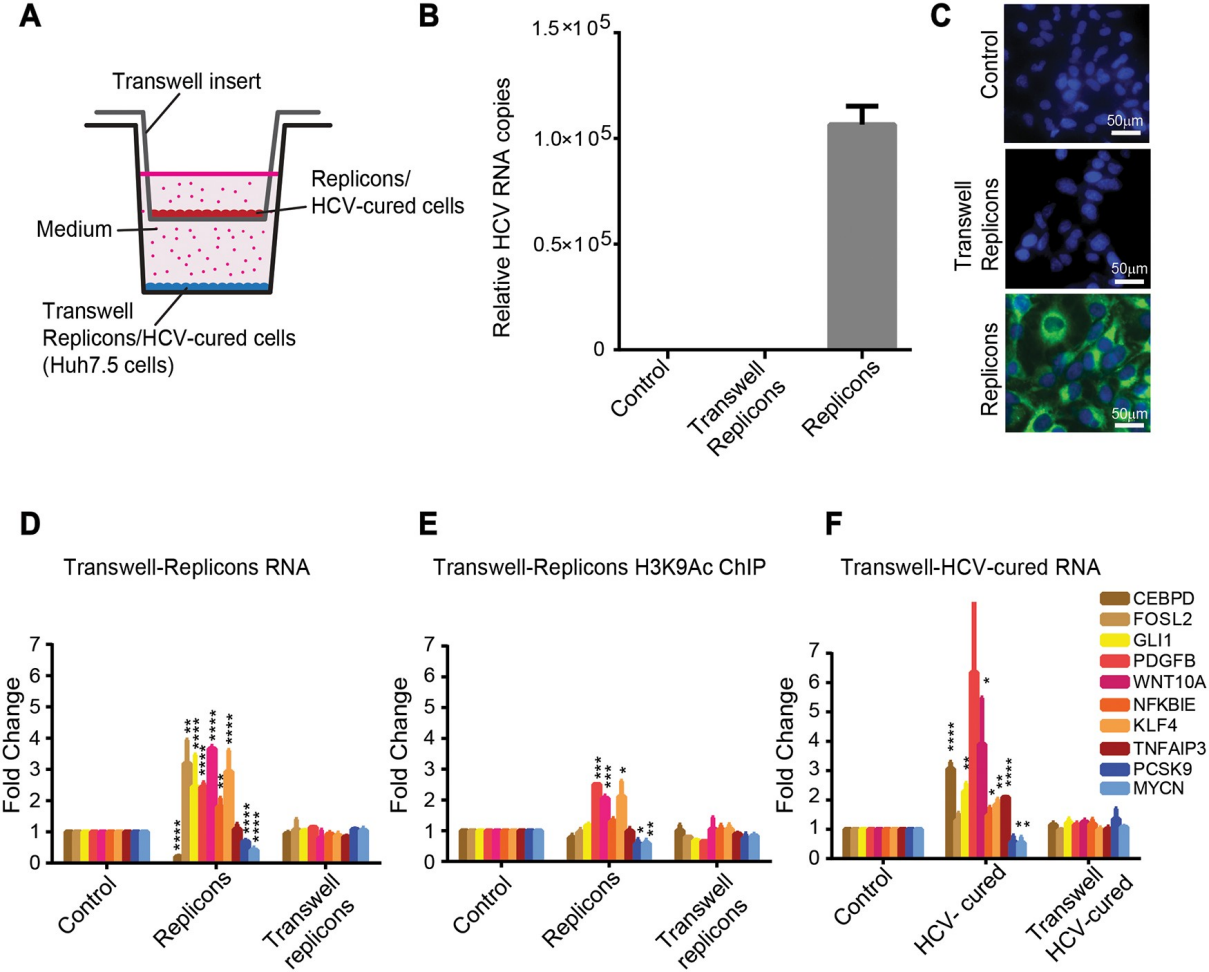
HCV-induced epigenetic alterations require the presence of HCV RNA or/and the expression of viral proteins in the cells. (A) Huh7.5 cells were seeded in a 12 well plate. Inserts were placed on top of the wells in the plate and replicon cells were seeded in the upper compartments of the Transwell inserts. Cells were incubated for 7 days with free diffusion of the medium through the insert’s pores. Then, the RNA from cells in both upper and lower compartments was extracted and qPCR was performed for the tested genes. (B) Levels of HCV RNA were quantified by qRT-PCR using primers for the HCV RNA 3’ UTR in replicon cells, compared to transwell replicon and control cells. Relative HCV RNA copies are calculated for replicon cells compared to control cells per ng of total cellular RNA. Differential expression was calculated using the equation of 2^(-ΔΔCt)^, with the GAPDH as an endogenous control. Means mRNA levels of HCV are shown ± SD from three independent experiments. (C) Immunostaining to validate expression of HCV proteins in replicon cells. Replicon cells or transwell replicon cells or control cells were stained with HCV-positive serum and anti-human 488 Alexa fluor as secondary antibody and visualized by fluorescence microscopy. Scale bars: 50μm. (D) The relative quantity of mRNA in replicon cells in the upper compartment, or Huh7.5 cells in the lower compartment (transwell replicon cells) was calculated compared to Huh7.5 control cells as quantified by qRT-PCR with primers specific for the tested genes. Differential expression was calculated using the equation of 2(-ΔΔCt), with the GAPDH as an endogenous control. Means fold change of mRNA levels (calculated compared to Huh7.5 control cells) are shown ± SD from three independent experiments. (E) Fold change values for H3K9Ac ChIP from replicon cells in both compartments. The IP DNA level was quantified by qPCR with primers for specific genes. Values were normalized relative to qPCR for these genes following ChIP with normal Rabbit IgG Ab as control. Values representing average ± SD from three biological replicates. (F) The Transwell assay was repeated as above but with HCV-cured cells seeded at the upper compartment of the Transwell inserts. Means fold change of mRNA levels (calculated compared to Huh7.5 control cells) are shown ± SD from three independent experiments (*p<0.05, **p<0.01, ***p<0.001, ****p<0.0001, t-test).

We next determined whether the presence of HCV RNA and/or expression of the viral proteins in the cells is required to induce the epigenetic alterations we observed in infected cells, or whether these changes may be induced in a paracrine manner by soluble factors secreted from infected cells to the surrounding non-infected cells. To distinguish between these possibilities, we performed a Transwell assay. Wells were seeded with Huh7.5 cells (termed here "transwell replicons") and the second compartment was seeded with the HCV-N NS3-NS5B subgenomic replicon cell line that supports HCV RNA replication and expresses the viral non-structural proteins. The membrane separating the compartments allows the free movement of soluble factors (Fig 5A). Cells were maintained in the wells for one week since we observed that one week of virus infection is sufficient to allow the epigenetic changes to occur as described in all the above experiments. We confirmed expression of viral RNA by qPCR, (Fig 5B) and expression of viral proteins by immunostaining, both in replicon cells compared to control and transwell replicon cells (Fig 5C). Evaluation of mRNA levels of specific genes in both compartments by qPCR, compared to control Huh7.5 cells, revealed that while the expected changes of gene expression occurred in replicon cells, no change was observed in the transwell replicon cells grown in the same medium (Fig 5D). We also performed ChIP to evaluate H3K9Ac in both compartments and observed comparable changes in replicon cells, but not in transwell replicon cells grown in the same medium (Fig 5E). In addition, we repeated this experiment with HCV-cured cells, in which the HCV-induced epigenetic signature maintains the altered gene expression, and again observed no change in the control cells grown in the same medium (termed here "transwell HVC-cured cells") (Fig 5F). We did not perform this experiment with HCV infected cells since the secreted virus would infect the non-infected cells seeded in the other compartment that shares the same medium. This experiment provides evidence supporting the requirement for the presence of HCV RNA and/or expression of viral proteins in the cells for the induction of the epigenetic and transcriptional alterations.

### The HCV-induced epigenetic signature can be reverted by specific inhibitors

**Fig 6:**
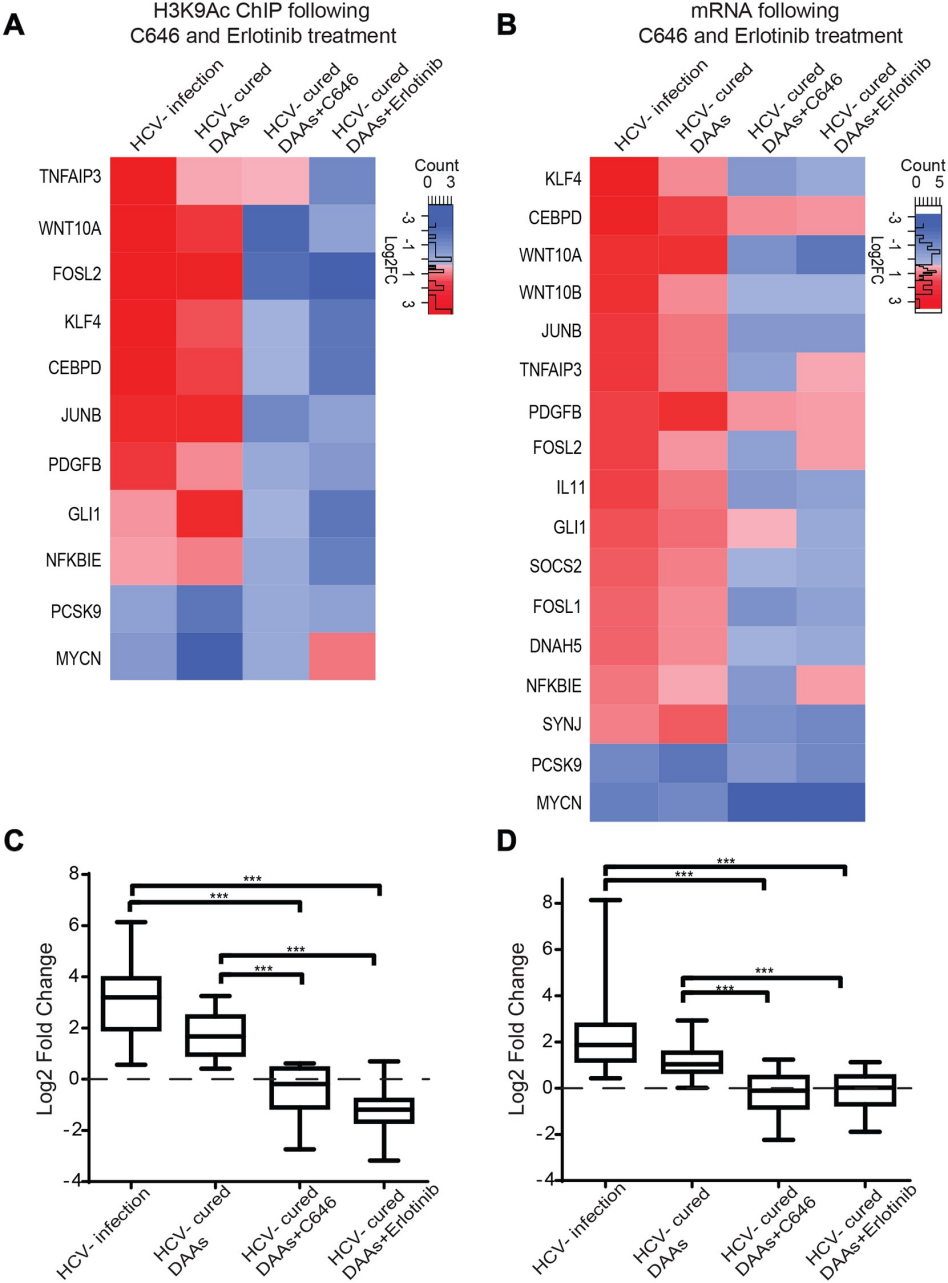
The HCV-induced epigenetic signature is reverted following treatment with specific inhibitors. (A) Cured and control cells (non-infected Huh7.5 cells also treated with DAAs) were treated with 10 μM C646 or 1μM of EGFR inhibitor erlotinib for 1 week. Following treatment, H3K9Ac ChIP was performed on C646/erlotinib treated cured cells (normalized to control DAAs treated and then C646/erlotinib treated cells) compared to DAAs treated cells in the absence of C646/erlotinib (normalized to control DAAs treated cells), and to HCV infected cells (normalized to non-infected cells). The H3K9Ac level was quantified by qPCR with primers for specific genes. Values were normalized relative to qPCR for these genes following ChIP with normal Rabbit IgG Ab as control. The means ± SD of Log2 fold change values from three biological replicates are presented as a heatmap. (B) Cured and control cells were treated with 10 μM C646 or 1μM erlotinib for 1 week as described above. Following treatment, RNA was purified and qRT–PCR using primers for specific genes was performed. The means ± SD of Log2 fold change values from three biological replicates are presented as a heatmap. (C and D) Box plots presenting the mean of Log2 fold change values of H3K9Ac (C) or mRNA (D) levels for the tested genes (only upregulated) for each treatment: HCV infected, HCV-cured by DAAs, HCV-cured by DAAs+C646, HCV-cured by DAAs+erlotinib (***p<0.001).

Since epigenetic alterations are potentially reversible, we tested whether inhibitors for epigenetic modifying enzymes could revert the HCV-induced epigenetic signatures. As the most significant epigenetic changes following infection were observed for the epigenetic marker H3K9Ac, we evaluated the reversion of the altered levels of H3K9Ac by using histone acetyl transferase (HAT) p300/CBP inhibitor C646 that inhibit acetylation of H3K9. HCV cured cells were treated with the non-cytotoxic concentration (S9 Fig) of inhibitor C646 for 7 days. ChIP analysis was performed for the H3K9Ac marker, and the mRNA levels of specific genes that correlated with altered H3K9Ac level were analyzed by qPCR. The log2 fold change in H3K9Ac and mRNA levels were calculated compared to control cells treated as the tested cells. Treatment with the inhibitor reverted the changes in H3K9Ac level (Fig 6A and S10A Fig) and gene expression level (Fig 6B and S10B Fig) observed in HCV infected and cured cells, except for the gene *MYCN*. The mean levels of H3K9Ac (Fig 6C) or mRNA (Fig 6D) for the tested genes (only upregulated) in HCV infected and HCV cured cells significantly differ following treatment with C646 that reverted these levels comparable to the controls (p<0.0001). These results verify the dependence of the expression of these genes on H3K9Ac levels, and suggest that epigenetic inhibitors may revert epigenetic signatures induced by HCV.

Although epigenetic drugs may demonstrate efficient reversion of the epigenetic alterations, they are non-specific, their effect is genome wide and they are not targeted specifically to the alterations induced by the virus. Therefore, we sought to evaluate the effect of a more specific inhibitor that targets a host pathway known to be altered by the virus. According to our data, the most significant pathways we identified to be epigenetically altered by HCV involve cytoskeleton remodeling (Fig 3) which is related to invasion and metastasis of cancer cells [31]. The epidermal growth factor receptor (EGFR) signaling pathway is involved in cancer invasion and metastasis and regulation of gene expression, and is a prime target for the treatment of malignant cancers [32]. EGFR is a major factor in cirrhosis and HCC. Over expression of this factor is associated with increased fibrosis and progression of cirrhosis and elevated risk of developing HCC [33]. Importantly, erlotinib, a specific EGFR inhibitor, reduces the expression of several EGFR ligands and also can reverse cirrhosis gene signature [33]. HCV was shown to specifically activate the EGFR signaling pathway [34]. In addition, EGFR is involved in HCV entry, and thus erlotinib inhibits HCV infection [21]. Previous study reported that EGFR activation trigger phosphorylation of p300 by protein kinase B (AKT) and this phosphorylation promotes the HAT activity of p300 to regulate histone acetylation (14). Considering all the above, we sought to evaluate whether activation of EGFR pathway by HCV may be a mechanism by which the infection induce epigenetic changes, and whether consequently, erlotinib will revert the epigenetic signature. Indeed, for all of the tested genes, treatment with erlotinib reverted the level of epigenetic marker H3K9Ac, compared to HCV infected and cured cells (Fig 6A and S10A Fig). The mRNA levels were also reverted in erlotinib treated cells, except for the genes *FOSL2*, *NFKBIE* and *MYCN* (Fig 6B and S10B Fig). The mean levels of H3K9Ac (Fig 6C) or mRNA (Fig 6D) for the tested genes (only upregulated) in HCV infected and HCV cured cells significantly differ following treatment with erlotinib that reverted these levels comparable to the controls (p<0.0001). To strengthen these results, pointing for the role of EGFR activation by HCV in altering H3K9Ac levels and consequently gene expression, we have tested whether activation of EGFR by its ligand EGF correlatively induces changes in the expression of the tested genes. Treatment with EGF altered gene expression, correlating with the gene expression altered following HCV infection, except for the genes *CEBPD*, *PCSK9* and *MYCN* (S10C Fig). Interestingly, in concurrence, the mRNA levels for these genes were also not efficiently reverted in cured cells, following treatment with erlotinib (Fig 6B and S10B Fig).

## Discussion

The new interferon-free DAAs regimes constitute a landmark in the treatment of chronic HCV infections. With approximately 71 million chronic HCV infected patients worldwide that are candidates for treatment with DAAs, the number of SVR patients is expected to dramatically increase in the near future [2,4,7]. Now, few years into the DAAs era, it is possible to evaluate their effect on HCC incidence. The surprising remaining high risk for HCC development following SVR in patients treated with DAAs raises an intriguing question regarding the mechanisms that maintain HCC risk in these "cured" patients [6,7,9]. Elucidating these mechanisms has important clinical implications and may point for a potential solution for this cancer association. Our study provides a mechanistic link to these observations, demonstrating an epigenetic signature that is induced specifically by HCV infection and is maintained following virus eradication by DAAs. This is possible due to the plasticity of the epigenome that may be reprogramed in response to environmental cues. The new epigenetic state can be imprinted and maintained in subsequent cell divisions, even following the removal of the environmental cue, leaving an "epigenetic signature” due to molecular "memory" mechanisms that are not well understood [26]. Different models for maintaining the position of specific modifications on histones that were suggested are generally based on the marking of the specific genomic loci by nascent histones containing specific modification for epigenetic "writers", i.e epigenetic enzymes (as HATs), that reestablish these modifications in that region. In these models, maintaining the epigenetic state requires the continues actions of the epigenetic enzymes (reviewed in [26,35]). Such a signature was demonstrated by El-Osta and others in hyperglycemia-driven patterns, which remained stable, despite removal of the glycemic insult that drove the progression of diabetic complications [36–38]. Similarly, our observations suggest that once epigenetic changes have occurred following infection, the persistent HCV-specific gene expression pattern is maintained in cured cells and the presence of the virus is therefore no longer required for its oncogenic effects on the host cells. We have also suggested that this persistent effect may be linked to HCC progression, although in a relatively small cohort of HCV-cured patients. This viral "hit and run" scenario that we revealed may lay the mechanistic groundwork for further larger clinical studies assessing the role of the persistent HCV-induced epigenetic signature in residual HCC risk in SVR patients.

**Fig 7:**
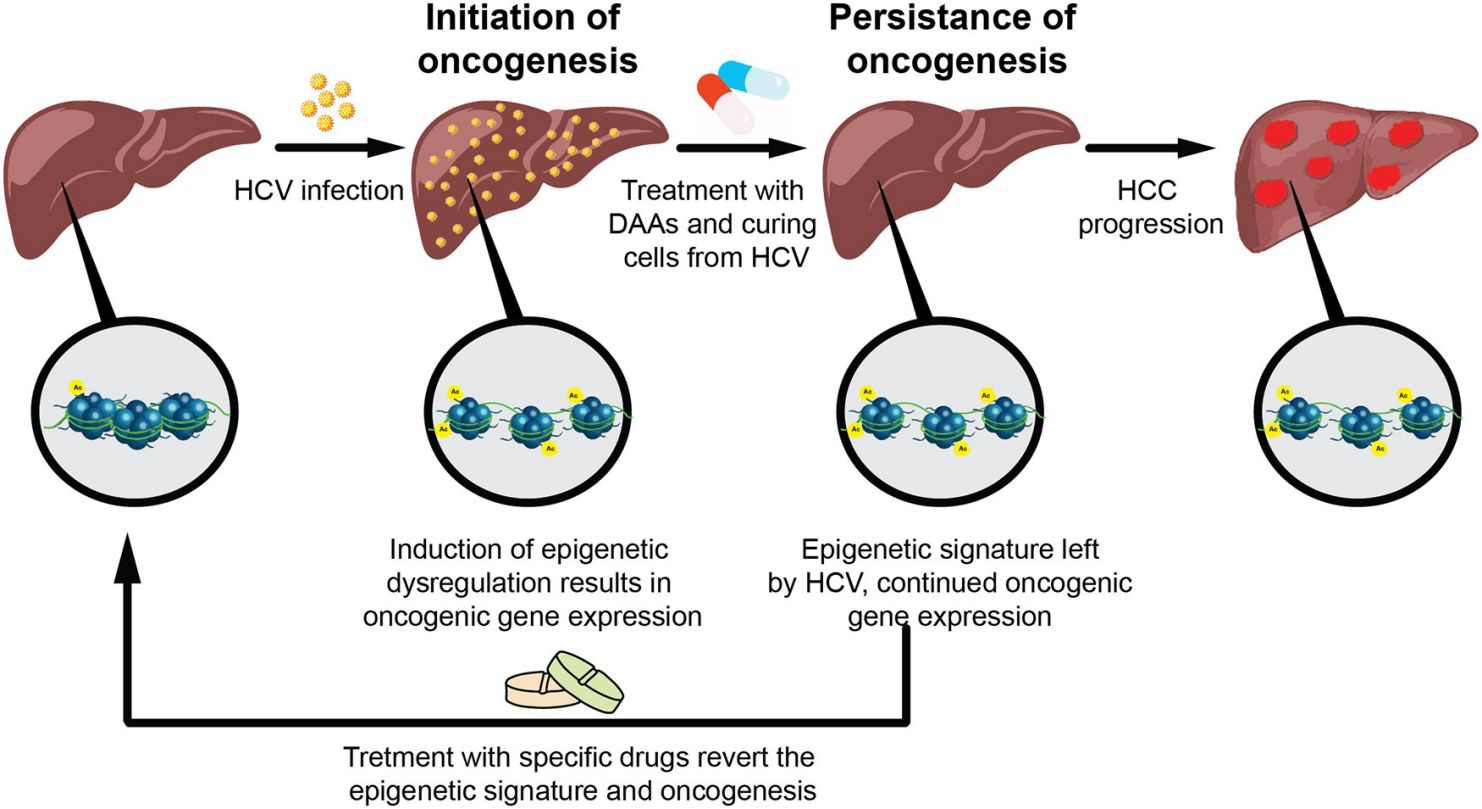
A model for HCV-induced oncogenesis after cure with DAAs.

Since the maintenance of epigenetic state, i.e. "epigenetic memory", requires the action of epigenetic enzymes, drugs that inhibit their function may antagonize the maintenance of epigenetic memory, thus enabling pharmacological reversion of dysregulated epigenetic states, such as in cancer [39]. Here we suggest that epigenetic inhibitors may revert the HCV-induced epigenetic signature. Moreover, we propose to evaluate the use of inhibitors that target specific pathway, such as the EGFR inhibitor erlotinib, that is altered by the virus and consequently induces the epigenetic changes following the infection. This approach may be more specific and prevent off-target effects, as compared to the genome wide activity of epigenetic drugs. Moreover, it may have important implications for assessing the use of these inhibitors to prevent progression to liver cancer in HCV-cured patients. This "hit and run" model for HCV pathogenesis and its potential reversion is illustrated in Fig 7.

In contrast to the DAAs treatment, with the interferon treatment we did not observe similar persistence of epigenetic signature. This finding is in concordance with recent evaluations that HCC development and recurrence may be more frequent following DAAs treatment compared to interferon-based treatment [2]. Therefore, the evaluation of the effect of interferon treatment on HCV-induced epigenetic changes should be further tested in clinical settings, and may open the question whether used of interferon treatment should be reevaluated.

Another important question regarding HCV pathogenesis is its effects on host signaling pathways. Recent reports demonstrated that HCV infection alters the DNA methylation state of the liver cells [40,41]. Here we demonstrate the ability of HCV to reorganize the distribution of histone PTMs genome-wide that alters host gene expression program. A complex map of networks of known and novel host factors that are epigenetically controlled by the virus is revealed. These alterations play an important role in its life cycle. The most significant pathways include cytoskeleton remodeling and the clathrin coated vesicle cycle. Cytoskeleton remodeling by reorganization of actin structure is required for transport of clathrin coated vesicles and both pathways play a vital role in all aspects of the viral life cycle including entry into the host cell, genome translation and replication, virion assembly and release from the cell [6,15,42]. Significant enrichment in factors involved in membrane re-organization is not crucial only for virus endocytosis and for egress from the cells, but also for the "membranous web" it produces for its replication and translation [43–45]. It is known that all stages of the virus life cycle are also tightly associated with lipids, and that the virus affects lipid metabolism [15,46,47]. Previous reports also demonstrated that HCV affects the host cell cycle [48,49]. We show that these pathways that were previously associated with pathogenesis and HCC [35,49,50], are epigenetically controlled by the virus. Importantly, we demonstrated the association between the altered expression of specific genes involved in these pathways and the increased risk for HCC development. These observations reveal novel insight into virus-host interactions and its link to viral pathogenesis.

In summary, this study provides a comprehensive profile of HCV-induced epigenetic alterations and demonstrates that HCV infection alters epigenetic markers thereby reprograming gene expression playing part in molecular pathways that affect the virus life cycle and are implicated in HCC development. Most importantly, these epigenetic changes persist as an epigenetic signature following virus eradication with DAAs treatment. Taken together, our findings may have implications for the clinical management of HCC. They suggest novel therapeutic approaches, to erase the cancer-associated gene expression signature that remains following viral eradication.

## Materials and methods

### Ethics statement

All human tissues were collected using protocols approved by the Institutional Review Boards of the Rabin Medical Center in Israel and the Kaohsiung Medical University in Taiwan and in accordance with the ethical standards of the Helsinki Declaration. Written consents were obtained from the patients.

### Cell lines

Huh-7.5 cells (a generous gift from Charles Rice, Rockefeller University) and Huh7/FT3-7 cells (a generous gift from Stanley M Lemon, University of North Carolina at Chapel Hill) were cultured as described previously [51]. To generate hepatocyte-like cells (termed herein Huh-7.5-HS), Huh-7.5 cells were maintained in 2% human serum for 2 to 4 weeks, as previously described [24,25]. The Clonetics Hepatocyte Cell Culture System containing normal primary human hepatocytes (PHH) (Lonza) were cultured as described previously [52]. The HCV-N NS3-NS5B subgenomic replicon cell line included in this study was cultured as we previously described [53].

### Virus

The HJ3–5 is H77C/JFH1 chimeric virus containing core-NS2 proteins from genotype 1a and NS3-NS5B from genotype 2a (a generous gift from Stanley M. Lemon, University of North Carolina at Chapel Hill (39)). The other chimeric viruses contain core-NS2 proteins from genotypes 2–7: J6/ JFH1 2b, S52/ JFH1 3a, ED43/ JFH1 4a, SA13/ JFH1 5a, HK6A/ JFH1 6a, QC69/ JFH1 7a (a generous gift from Jens Bukh (41)). Virus stocks were produced in Huh7/FT3-7 cells and viral titers were determined by FFU assay in Huh-7.5 cells, as described previously (39). For all experiments with Huh7.5 or Huh7.5-HS, cells were infected with HJ3-5 chimeric virus at a MOI of 0.1 or 0.5 and passaged for at least 2 weeks until approximately 100% of the cells were HCV positive, determined by immunofluorescence, as previously described [53]. PHH (Lonza) were infected with cell culture infectious HCV HJ3-5 at high MOI (0.5–1) to ensure high efficiency of infection, as described previously [52]. PHH cells were harvested after 1 week in culture, when most of the cells were infected.

### Resected liver tissue samples

Resected liver tissues were obtained from the Rabin Medical Center in Israel and the Kaohsiung Medical University in Taiwan. In total we collected 17 liver biopsies that include: 7 HCV-infected biopsies (No. 1–7), 4 SVR biopsies following DAAs treatment (No. 8–11) and 6 control biopsies obtained from normal liver tissues (No. 12–17). The clinical data for these patients is presented in S4 Table. All samples were cryopreserved before used for ChIP assays and qPCR.

### ChIP and ChIP-seq

ChIP assays were performed using the Magna Chip G kit (Milipore) according to the manufacturer’s instructions. 100% HCV infected (generated as described above) and non-infected Huh7.5 cells were fixed with 1% formaldehyde to cross-link proteins to DNA. Cells were then lysed, and chromatin was fragmented by sonication (Covaris S220) to generate DNA fragments of 200–1000 bp. Chromatin was incubated overnight with either specific antibodies against H3K9Ac, H3K43Me, H3K93Me and H3K273Me (Millipore) or normal Rabbit IgG Ab (Milipore) as a negative control for the immunoprecipitation experiments. Cross-links were reversed, and DNA purified.

Libraries for next generation sequencing (NGS) were prepared by using NEBNext Ultra TM DNA Library Prep Kit for Illumina (NEB) and sequenced using Illumina HiSeq 2500 platform with single-end reads of 50 bp according to the manufacturer’s instructions. For each ChIP, at least three biological replicates were performed. For resected liver tissues: ChIP assays were performed using the iDeal ChIP seq kit for Histones (Diagenode) according to the manufacturer’s instructions. For each sample, 20–40 mg of liver tissue was chopped, homogenized, and subjected to ChIP using H3K9Ac or a Rabbit IgG Ab as negative control (Diagenode).

### RNA-seq

Total RNA from 100% HCV infected (generated as described above) and non-infected Huh7.5 cells was purified using the RNeasy Mini Kit (Qiagene). Next, 1 μg of total RNA was treated with the NEBNext poly (A) mRNA Magnetic Isolation Module (NEB). RNA-seq libraries were produced using the NEBNext Ultra RNA Library Prep Kit for Illumina (NEB). For each tested group six biological replicates were performed. All libraries were sequenced by Illumina HiSeq 2500 platform with single-end reads of 50 bp, according to the manufacturer’s instructions.

### Bioinformatic analysis

#### ChIP-seq bioinformatic analysis

Short sequence tags were quality trimmed, and sequences shorter than 35 nt were discarded. The sequences were aligned to the GRCh37 (hg19) reference genome using BWA [54]. Duplicate alignments were removed. Peaks were generated for each sample using MACS [55] with an input sample used to define the background. Peaks were merged, and any region covered by at least two sample-based peaks was used for downstream differential analysis. Aligned reads within the consensus peak regions were counted using BEDtools multicov [56] with a minimum alignment quality threshold of 20. Differential analysis of infected versus non-infected cells was performed using edgeR [57].

#### RNA-seq bioinformatic analysis

Short sequence tags were quality trimmed, discarding sequences shorter than 35 nt. The sequences were aligned to the GRCh37 (hg19) reference genome using STAR [55]. Aligned reads were counted using featureCounts [58], by means of the Ensemble Version 75 transcript database. Differential analysis was performed using edgeR [57].

#### Integration between ChIP-seq and RNA-seq data

ChIP-seq peaks were assigned gene names based on their proximity to the nearest transcription start site (TSS) from the ChIP-seq data sets. Integrative analysis was then performed using the gene names to join the ChIP-seq and RNA-seq data sets.

#### Pathway analysis

To identify the significantly affected biological processes, sets of up- and down-regulated genes were analyzed by multiple bioinformatic tools. GeneGo: The pathway analysis was generated from integrated RNA-seq and ChIP-seq data. Using the RNA-Seq and H3K9Ac ChIP-Seq data we generated ranked lists of differentially modified genes (DMGs, for ChIP-seq signal at a vicinity of a specific gene; for peaks within gene body, both exons or introns, and within TSS, up to 5kb upstream of the TSS) and differentially expressed genes (DEGs, for RNA-seq signal) (Ranked from most significant decrease to the most significant increase). The two gene lists were used for gene ontology (GO) analysis using the MetaCore database from Thomson Reuters (ver. 6.11, build 41105, GeneGo, Thomson Reuters, New York, NY, USA) [29] pathway analysis tool. This pathway analysis tool was used to perform gene network enrichment analysis from integration of RNA-seq and H3K9Ac ChIP-seq data. All genes that were identified as DMGs or DEGs were included in the analysis. The enrichment analysis consisted of matching gene IDs of possible targets for the "common”(black/white bar), and "unique" (RNA-seq-black, H3K9Ac ChIP-seq-grey) sets with gene IDs in functional ontologies in MetaCore. The probability of a random intersection between a set of IDs the size of the target list with ontology entities is estimated by the p value of the hypergeometric intersection. The lower p value reflects higher relevance of the entity to the dataset, which shows a higher rating for the entity. A canonical pathway represents a set of signaling and metabolic maps. These are created based on published peer reviewed literature using the Thompson Reuter MetaCore analysis, GeneGo. The top scored maps (map with the lowest p value) are based on the enrichment distribution sorted by the ‘common’ set.

Correlation of GO gene sets with TSS: Profiles of the combined H3K9Ac Chip-seq data were created across the TSS regions of genes belonging to the indicated GO gene sets. Chip-seq reads were summed into 100bp bins +/- 4 kbs from TSS, then normalized by total aligned reads. As control background, the TSS regions of 1000 random genes were analyzed.

Gene Set Enrichment Analyzes (GSEA). A ranked gene list was generated for the differential H3K9Ac ChIP-seq data according to the calculated p value. This ranked list was used for Gene Set Enrichment Analysis (http://software.broadinstitute.org/gsea/index.jsp).

#### Quantitative RT-PCR and PCR

Total RNA from tested cells was extracted using a RNA kit (Qiagene). Equal amounts of the RNA isolated from treated or control cells were transcribed into cDNA using the High Capacity cDNA Reverse Transcriptase kit (Applied Biosystems) and analyzed by RT-PCR using Power SYBR Green Master Mix (Life Technologies). Oligonucleotides were designed using the Primer3 PCR Primer Design Tool according to the RNA-Seq and ChIP-seq data. Primers are listed in S5 Table. The thermal program included 10 min incubation at 95° followed by 40 cycles of 95° for 10 s, 58° for 10 s and 72° for 20s. Differential expression was calculated using the equation of 2^(-ΔΔCt)^, with GAPDH as an endogenous control. RT-PCR analysis was conducted using RNA from three independent experiments. For validation of ChIP-seq data, primers for qPCR were designed to target the peaks of the tested genes according to the ChIP-seq data. Purified DNA following ChIP was quantified by qPCR. Values were normalized relative qPCR for these genes following ChIP with normal Rabbit IgG Ab as control. For quantifying HCV RNA, real time qPCR was carried out using primers for the 3′-untranslated region (UTR) of HCV.

#### Cell viability assay

Cell viability was tested by XTT assay as previously described [59].

#### Treatment of HCV infected cells with antivirals

The all oral interferon-free FDA approved anti-HCV DAAs combination contain: dasabuvir (NS5B nonnucleoside polymerase inhibitor), ombitasvir (a NS5A inhibitor), paritaprevir (NS3/4A protease inhibitor) and ritonavir (pharmacokinetic (PK) enhancer) [27,28]. This DAAs combination was diluted in DMSO. The optimal final concentration of DAAs for all experimental procedures was determined following XTT assay and the optimal mixture contained 0.4 μM dasabuvir, 0.03 μM ritonavir, 0.05 μM paritaprevir 0.007 μM ombitasvir diluted in DMEM. Huh7.5 cells were infected with HJ3-5 chimeric virus at a MOI of 0.1 and passaged for 2 weeks until approximately 100% of the cells were HCV positive. Following viral infection, cells were treated with DAAs for 7 days. During the therapy period and every week for 2 months post treatment, cells were immunostained with HCV-positive serum and anti-human 488 Alexa fluor (Jackson) as secondary antibody to confirm that there is no HCV infection. Infection was visualized by fluorescence microscopy. For curing the cells with interferon, 15ng/ml of interferon α 2a (Sigma) was used. Huh7.5 cells were infected, treated for 3 weeks until no viral RNA or proteins were detected in the cells, and monitored as described above.

#### Treatment of HCV-cured cells with inhibitors

To evaluate the reversion of the epigenetic signature by inhibitors, HCV-cured cells and control cells were treated with 10μM of C646 or 1μM of the EGFR inhibitor erlotinib (LC labs) for 1 week, before analysis by qPCR or ChIP-qPCR using primers for specific genes (see S5 Table for primers list).

#### Treatment of cells with EGF

Huh7.5 cells were seeded in a 12 well plate and grown for 24 hr without serum. Then, 50ng/ml of EGF was added to the cells. After 48hr, cells were harvested and qPCR was performed as described above.

#### Transwell assay

Huh7.5 cells were seeded in a 12 well plate. Inserts were placed on top of the wells in the plate and replicon cells were seeded in the upper compartments of the Transwell inserts. Cells were incubated for 7 days with free diffusion of the medium through the insert’s pores. Then, the RNA from cells in both upper and lower compartments was extracted and qPCR was performed for the tested genes. The relative quantity of mRNA in replicon cells in the upper compartment, or Huh7.5 cells in the lower compartment (transwell replicon cells) was calculated compared to Huh7.5 control cells as quantified by qRT-PCR with primers specific for the tested genes. ChIP-qPCR experiment to evaluate the level of H3K9Ac in cells in both upper and lower compartments was also performed as described above.

#### Patient survival analysis

Microarray-based genome-wide profile data was obtained from the NCBI Gene Expression Omnibus (GSE15654) [30]. The dataset included gene expression profiles of liver biopsies from 216 patients with hepatitis C-related Child-Pugh class A cirrhosis. The log-rank test and Cox regression modeling were used to evaluate association between gene expression and time from the enrollment to development of HCC using MATLAB (The Mathworks, Inc.) and R statistical package (www.r-project.org). Patient samples were split into high and low gene expressing groups based upon median Z-score cutoff of 0. The analysis included only samples within 10 years from enrollment.

#### Statistical analysis

Statistical significance was calculated using unpaired, two-tailed Student’s *t*-test. Values were considered statistically significant if the P value was < 0.05. For all figures, * indicates P value <0.05; ** indicates P value <0.01; *** indicates P value <0.001 and **** indicates P value <0.0001. Error bars represent SEM.

## Supporting information

S1 Fig Integration of RNA-seq with H3K9Ac and H3K4Me3 ChIP-seq data analysis. Overlap between H3K4Me3, H3K9Ac regions and mRNA regions in whole gene or TSS regions. Transcripts were matched to H3K4Me3 and H3K9Ac regions using BLASTN.
(PDF)

S2 Fig Validation of RNA-seq and ChIP-seq. Validation of H3K9Ac (A) and H3K4Me3 (B) ChIP-seq and RNA-seq (C) by qRT-PCR for specific genes in HCV infected cells normalized to non-infected cells. (D) Validation of control non-affected genes by qRT-PCR. Differential expression was calculated using the equation of 2^(-ΔΔCt)^, with the GAPDH as an endogenous control. For each ChIP-seq and ChIP-qPCR validation, three to five biological replicates were conducted and for RNA-seq and mRNA validation by qPCR six biological replicates were performed. Values were normalized relative to qPCR for these genes following ChIP with normal Rabbit IgG Ab as control.
(PDF)

S3 Fig Validation of gene expression in HCV-infected PHH. (A) Clonetics PHH were seeded on palates precoated with collagen and maintained according to the manufacturer’s instructions and as previously described [52]. Cultured PHH were infected with HCV at MOI 0.5–1 for 1 week. (A) Infected PHH cells were immunostained with HCV-positive serum and anti-human 488 Alexa fluor as secondary antibody. Infection was visualized by fluorescence microscopy. Scale bars: 20μm. (B) Levels of HCV RNA in HCV-infected PHH cells normalized to non-infected PHH cells as quantified by qRT-PCR with primers for the HCV RNA 3’ UTR. Shown are Log10 of relative HCV RNA copies calculated compared to non-infected PHH cells per ng of total cellular RNA. Differential expression was calculated using the equation of 2^(-ΔΔCt)^, with the GAPDH as an endogenous control. (C) Validation of differentially expressed genes in HCV-infected PHH compared to HCV-infected Huh7.5 cells, both normalized to non-infected cells.
(PDF)

S4 Fig Validation of gene expression in HCV-infected Huh7.5-HS. (A) Huh7.5 cells maintained in human serum were infected with HCV for up to 60 days. Levels of HCV RNA in HCV-infected Huh7.5-HS cells normalized to non-infected Huh7.5-HS cells as quantified by qRT-PCR with primers for the HCV RNA 3’ UTR, at 14, 42 and 60 days post infection. Relative HCV RNA copies are calculated compared to non-infected Huh7.5-HS cells per ng of total cellular RNA. Differential expression was calculated using the equation of 2^(-ΔΔCt)^, with the GAPDH as an endogenous control. (B) Validation of differentially expressed genes by qPCR in HCV-infected Huh7.5-HS cells for 14 days compared to 60 days both normalized to non-infected Huh7.5-HS cells. (C) Validation H3K9Ac ChIP for specific genes by qRT-PCR in Huh7.5-HS cells for 14 days compared to 60 days both normalized to non-infected Huh7.5-HS cells.
(PDF)

S5 Fig Gene expression profiling following infection with genotypes 1–7 chimeric HCVs. Huh7.5 cells were infected with chimeric viruses from genotypes 2–7. Infected cells were analyzed when approximately 100% of the cells were positive for HCV. (A) Levels of HCV RNA in the cells were quantified by qRT-PCR using primers for the HCV RNA 3’ UTR. Relative HCV RNA copies are calculated for Huh7.5 cured cells compared to non-infected Huh7.5 cells per ng of total cellular RNA. Differential expression was calculated using the equation of 2^(-ΔΔCt)^, with the GAPDH as an endogenous control. Log10 fold change of means mRNA levels of HCV are shown ± SD from three independent experiments. (B) Validation of differentially expressed genes in genotypes 1–7 HCV-infected Huh7.5 cells normalized to non-infected cells. Log2 fold change of means mRNA levels are shown ± SD from three independent experiments.
(PDF)

S6 Fig Evaluating the cytotoxicity of DAAs by XTT. Huh7.5 cells were incubated with DAAs in serial 1:5 dilutions to final concentrations as indicated in the table, for 72 hrs. The cell viability of Huh7.5 cells was assessed by the XTT assay. The XTT assay was measured at 500 nm with reference of 690 nm. In yellow marked the non-toxic concentration that was selected for future experiments.
(PDF)

S7 Fig Epigenetic alterations are reverted following cure of HCV by interferon. (A) HCV-infected and non-infected Huh7.5 cells were treated with 15ng/ml of interferon. RNA was purified from Interferon-cured cells and control interferon treated cells and qRT–PCR was performed using primers for specific genes. Log2 fold change values are also presented as heatmap; three biological replicates were performed. (B) H3K9Ac ChIP was performed on the Interferon-cured cells. The level of H3K9Ac for specific genes was quantified by qPCR, and values were normalized to those of interferon treated control cells. These levels were compared to HCV-infected cells and DAAs-cured cells. Log2 fold change values are also presented as heatmap; three biological replicates were performed.
(PDF)

S8 Fig GSEA generated from H3K9Ac ChIP-seq data. A ranked gene list was generated for the differential H3K9Ac ChIP-seq data according to the p value. This ranked list was used for Gene Set Enrichment Analysis (http://software.broadinstitute.org/gsea/index.jsp). Enrichment plots for significant gene sets are presented.
(PDF)

S9 Fig Evaluating the cytotoxicity of C646 by XTT assay. Huh7.5 cells were incubated with inhibitor in serial dilutions. The XTT assay was measured at 500 nm with reference of 690 nm.
(PDF)

S10 Fig The HCV-induced epigenetic signature is reverted following treatment with specific inhibitors. (A) Cured and control cells (non-infected Huh7.5 cells also treated with DAAs) were treated with 10 μM C646 or 1μM of EGFR inhibitor erlotinib for 1 week. Following treatment, H3K9Ac ChIP was performed on C646/erlotinib treated cured cells (normalized to control DAAs treated and then C646/erlotinib treated cells) compared to DAAs treated cells in the absence of C646/erlotinib (normalized to control DAAs treated cells), and to HCV infected cells (normalized to non-infected cells). The H3K9Ac level was quantified by qPCR with primers for specific genes. Values were normalized relative to qPCR for these genes following ChIP with normal Rabbit IgG Ab as control. The means ± SD of Log2 fold change values from three biological replicates are presented. (B) Cured and control cells were treated with 10 μM C646 or 1μM erlotinib for 1 week as described above. Following treatment, RNA was purified and qRT–PCR using primers for specific genes was performed. The means ± SD of Log2 fold change values from three biological replicates are presented for each gene. (C) Huh7.5 cell were treated with EGF for 48hr. Following treatment, RNA was purified and qRT–PCR using primers for specific genes was performed. The means ± SD of Log2 fold change values from three biological replicates are presented for each gene.
(PDF)

S1 Table Integration of changes in histone mark H3K4Me3 (ChIP-seq) and mRNA expression (RNA-seq) in HCV infected compared to non-infected cells for specific genes. (PDF)

S2 Table Integration of changes in histone mark H3K9Ac (ChIP-seq) and mRNA expression (RNA-seq) in HCV infected compared to non-infected cells for specific genes. (PDF)

S3 Table Pathway enrichment analysis. (XLS)

S4 Table Clinical and pathological features of patients. (PDF)

S5 Table List of primers. (XLSX)
