# Supplementary figures and images for: Hepatitis C virus leaves an epigenetic signature post cure of infection by direct-acting antivirals
Source: PLoS Genet. 2019 Jun 19;15(6):e1008181. doi: 10.1371/journal.pgen.1008181 (PMC6602261; doi:10.1371/journal.pgen.1008181)

Supplementary Figure 1

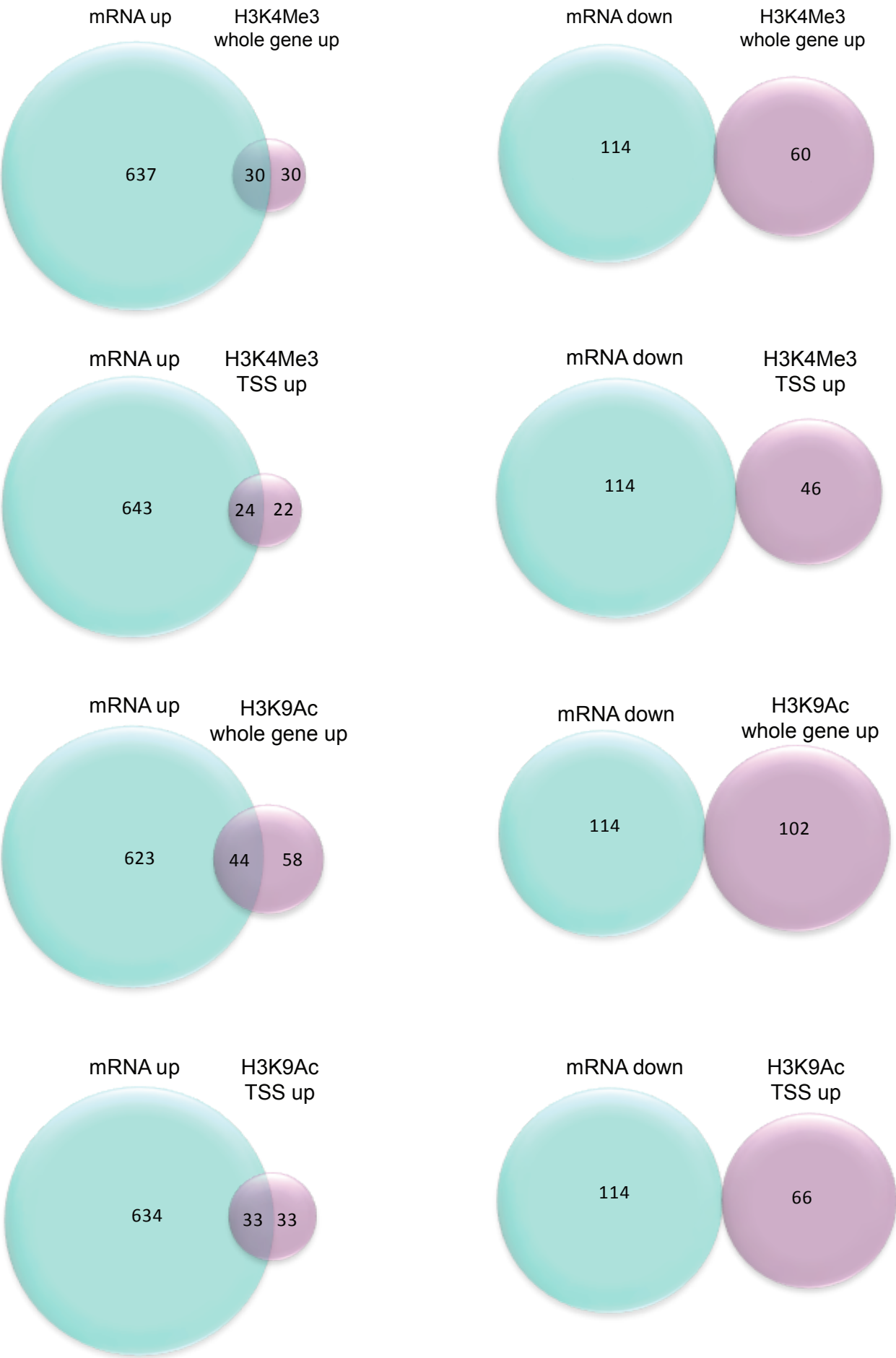

Supplement: S1 Fig — Overlap between H3K4Me3, H3K9Ac regions and mRNA regions in whole gene or TSS regions. Transcripts were matched to H3K4Me3 and H3K9Ac regions using BLASTN. (PDF) [file pgen.1008181.s001.pdf]

Supplementary Figure 2

A

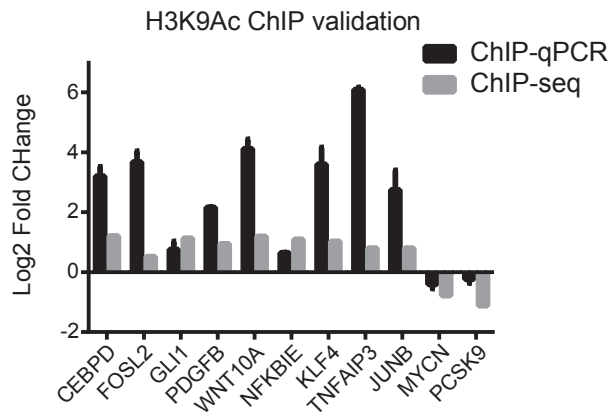

B

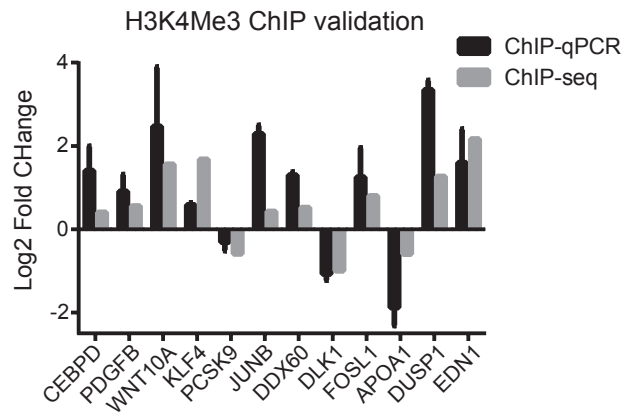

C

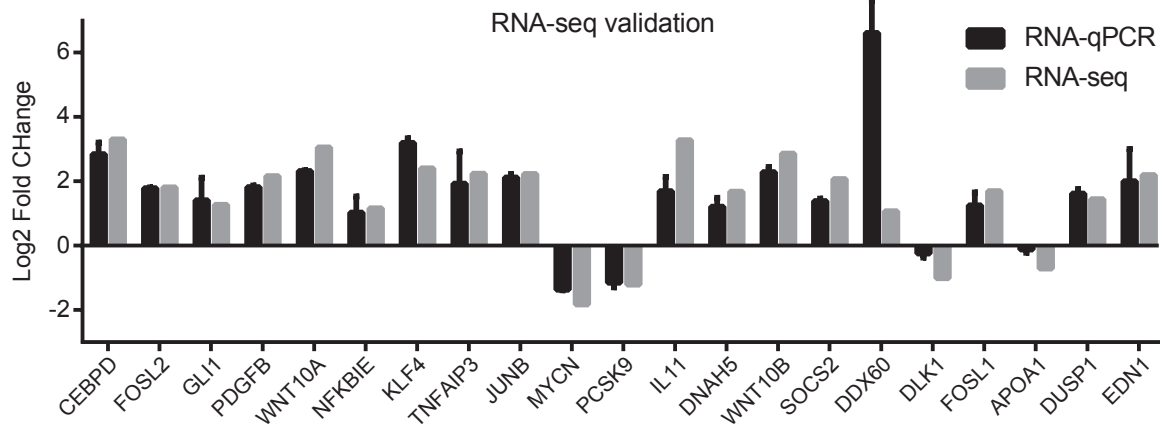

D

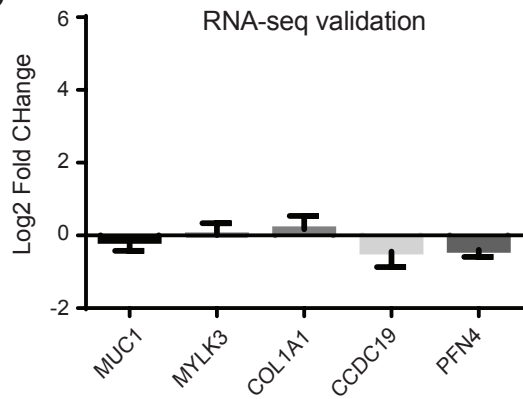

Supplement: S2 Fig — Validation of H3K9Ac (A) and H3K4Me3 (B) ChIP-seq and RNA-seq (C) by qRT-PCR for specific genes in HCV infected cells normalized to non-infected cells. (D) Validation of control non-affected genes by qRT-PCR. Differential expression was calculated using the equation of 2(-ΔΔCt), with the GAPDH as an endogenous control. For each ChIP-seq and ChIP-qPCR validation, three to five biological replicates were conducted and for RNA-seq and mRNA validation by qPCR six biological replicates were performed. Values were normalized relative to qPCR for these genes following ChIP with normal Rabbit IgG Ab as control. (PDF) [file pgen.1008181.s002.pdf]

Supplementary Figure 3

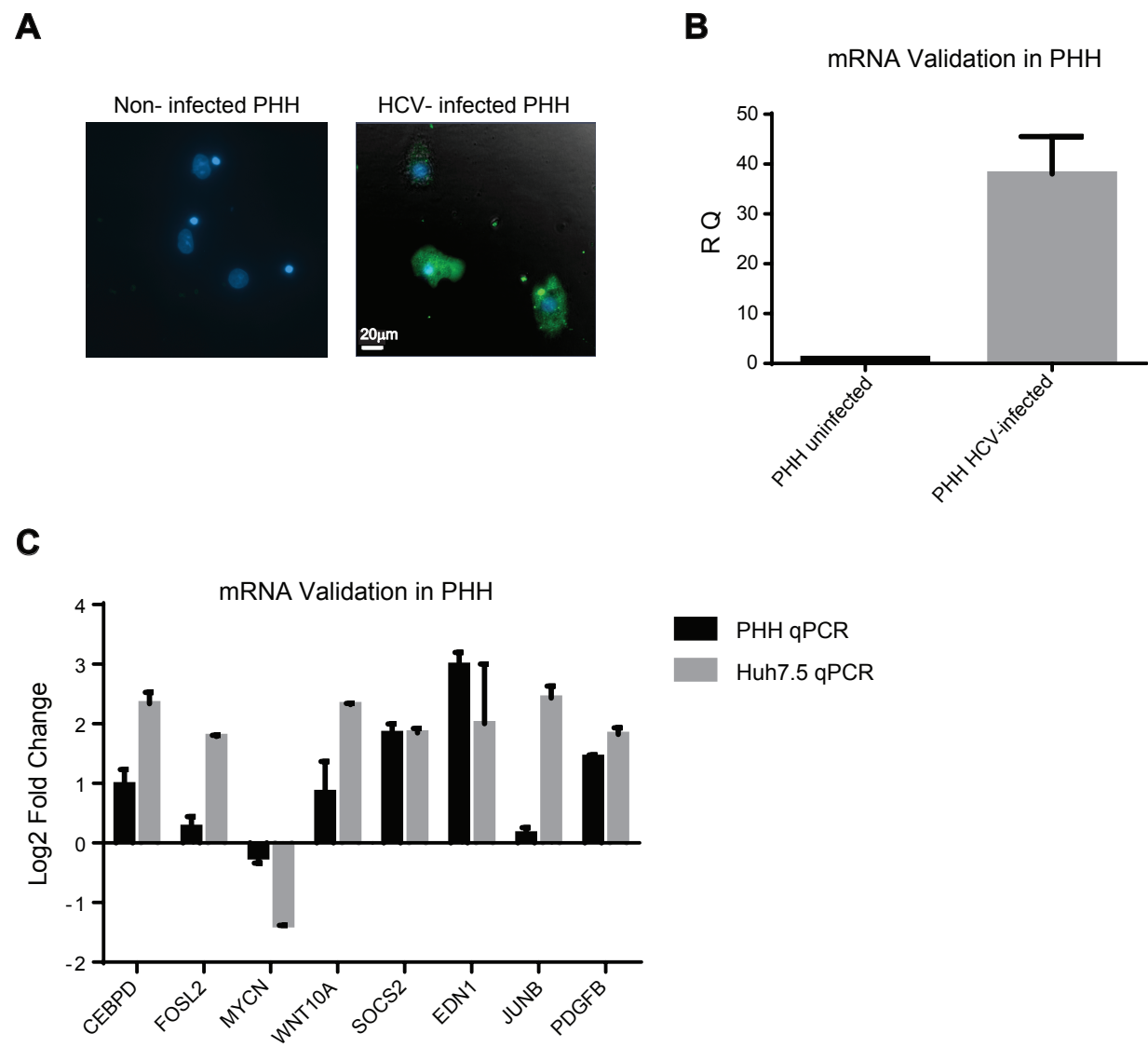

Supplement: S3 Fig — (A) Clonetics PHH were seeded on palates precoated with collagen and maintained according to the manufacturer’s instructions and as previously described [52]. Cultured PHH were infected with HCV at MOI 0.5–1 for 1 week. (A) Infected PHH cells were immunostained with HCV-positive serum and anti-human 488 Alexa fluor as secondary antibody. Infection was visualized by fluorescence microscopy. Scale bars: 20μm. (B) Levels of HCV RNA in HCV-infected PHH cells normalized to non-infected PHH cells as quantified by qRT-PCR with primers for the HCV RNA 3’ UTR. Shown are Log10 of relative HCV RNA copies calculated compared to non-infected PHH cells per ng of total cellular RNA. Differential expression was calculated using the equation of 2(-ΔΔCt), with the GAPDH as an endogenous control. (C) Validation of differentially expressed genes in HCV-infected PHH compared to HCV-infected Huh7.5 cells, both normalized to non-infected cells. (PDF) [file pgen.1008181.s003.pdf]

Supplementary Figure 4

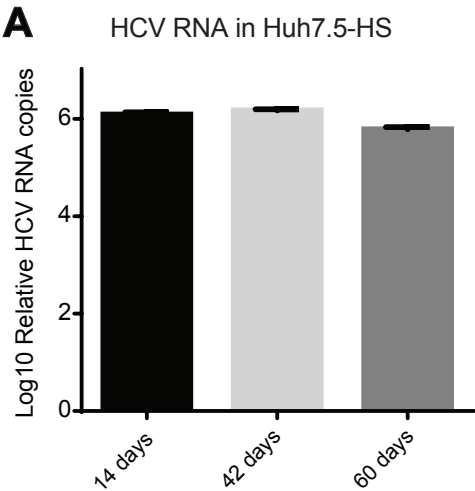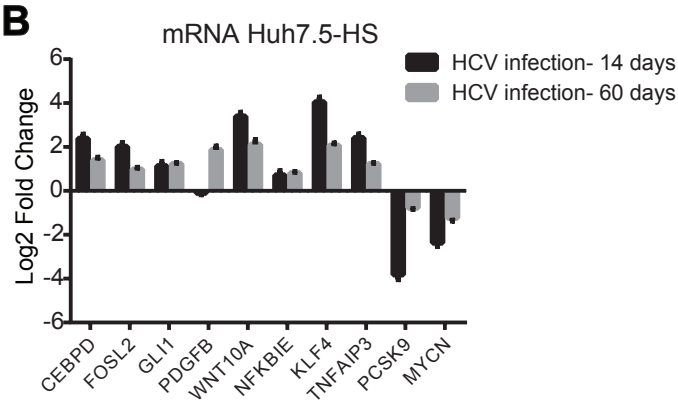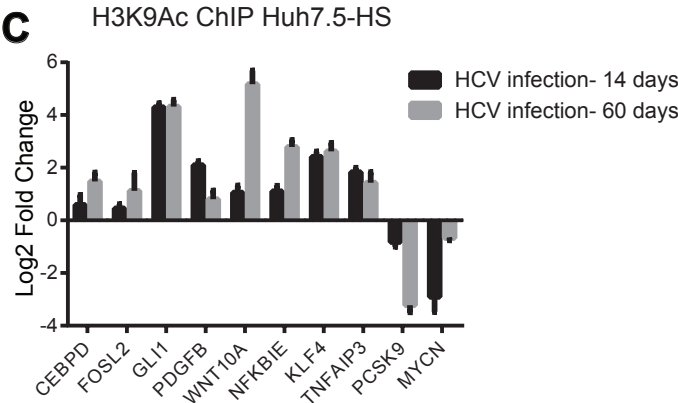

Supplement: S4 Fig — (A) Huh7.5 cells maintained in human serum were infected with HCV for up to 60 days. Levels of HCV RNA in HCV-infected Huh7.5-HS cells normalized to non-infected Huh7.5-HS cells as quantified by qRT-PCR with primers for the HCV RNA 3’ UTR, at 14, 42 and 60 days post infection. Relative HCV RNA copies are calculated compared to non-infected Huh7.5-HS cells per ng of total cellular RNA. Differential expression was calculated using the equation of 2(-ΔΔCt), with the GAPDH as an endogenous control. (B) Validation of differentially expressed genes by qPCR in HCV-infected Huh7.5-HS cells for 14 days compared to 60 days both normalized to non-infected Huh7.5-HS cells. (C) Validation H3K9Ac ChIP for specific genes by qRT-PCR in Huh7.5-HS cells for 14 days compared to 60 days both normalized to non-infected Huh7.5-HS cells. (PDF) [file pgen.1008181.s004.pdf]

Supplementary Figure 5

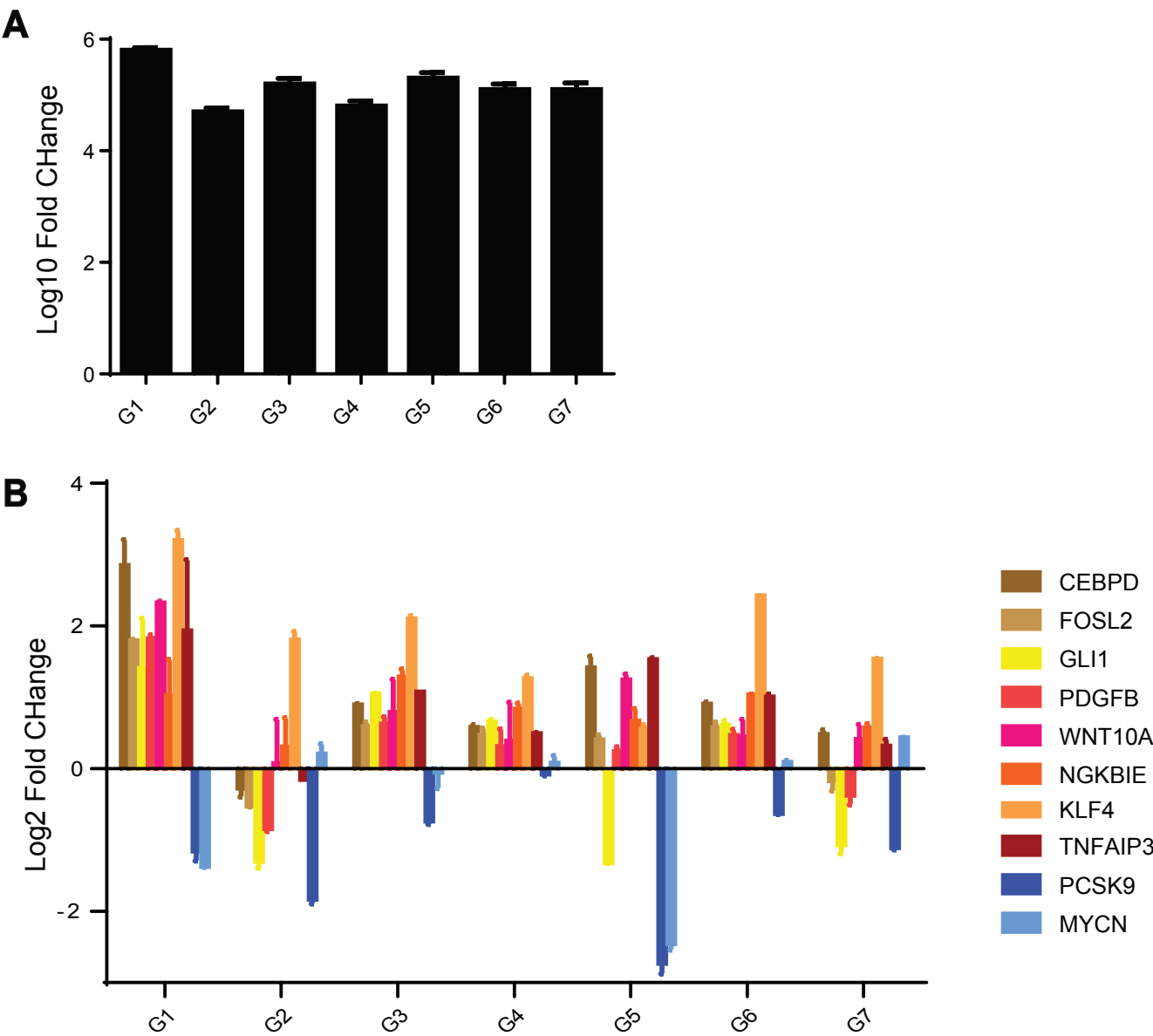

Supplement: S5 Fig — Huh7.5 cells were infected with chimeric viruses from genotypes 2–7. Infected cells were analyzed when approximately 100% of the cells were positive for HCV. (A) Levels of HCV RNA in the cells were quantified by qRT-PCR using primers for the HCV RNA 3’ UTR. Relative HCV RNA copies are calculated for Huh7.5 cured cells compared to non-infected Huh7.5 cells per ng of total cellular RNA. Differential expression was calculated using the equation of 2(-ΔΔCt), with the GAPDH as an endogenous control. Log10 fold change of means mRNA levels of HCV are shown ± SD from three independent experiments. (B) Validation of differentially expressed genes in genotypes 1–7 HCV-infected Huh7.5 cells normalized to non-infected cells. Log2 fold change of means mRNA levels are shown ± SD from three independent experiments. (PDF) [file pgen.1008181.s005.pdf]

### Supplementary Figure 6

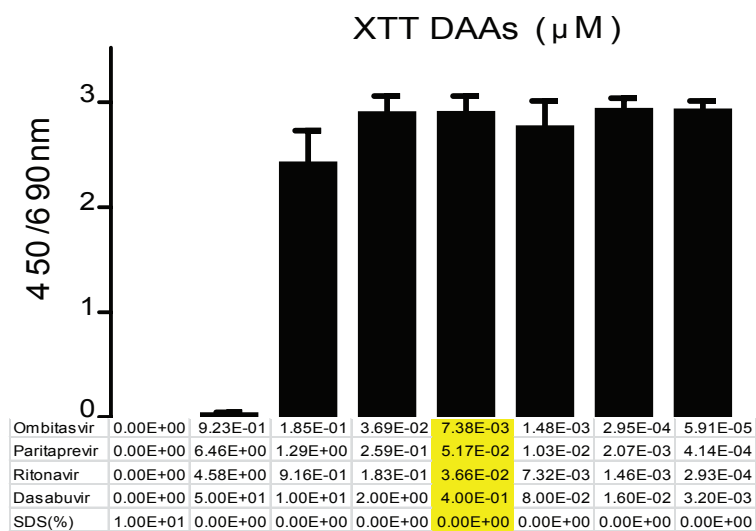

Supplement: S6 Fig — Huh7.5 cells were incubated with DAAs in serial 1:5 dilutions to final concentrations as indicated in the table, for 72 hrs. The cell viability of Huh7.5 cells was assessed by the XTT assay. The XTT assay was measured at 500 nm with reference of 690 nm. In yellow marked the non-toxic concentration that was selected for future experiments. (PDF) [file pgen.1008181.s006.pdf]

Supplementary Figure 7

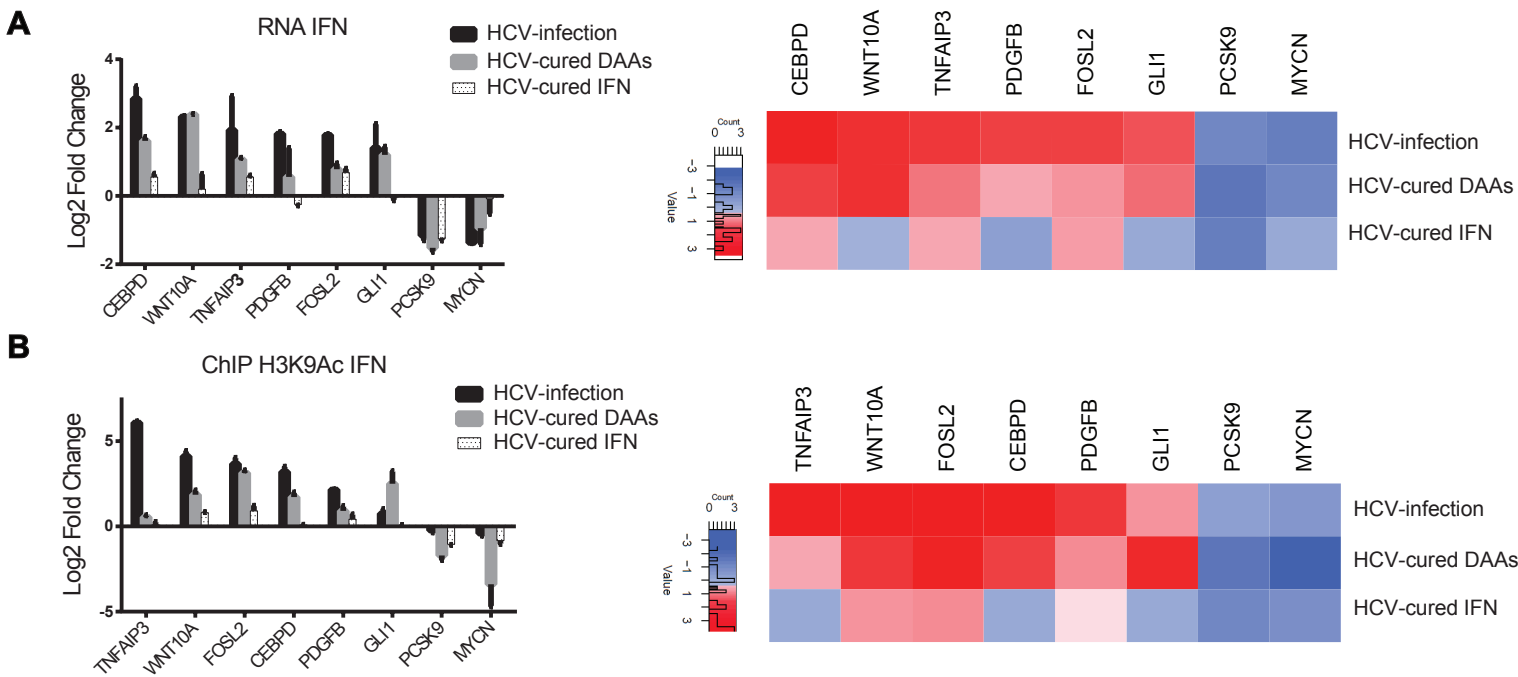

Supplement: S7 Fig — (A) HCV-infected and non-infected Huh7.5 cells were treated with 15ng/ml of interferon. RNA was purified from Interferon-cured cells and control interferon treated cells and qRT–PCR was performed using primers for specific genes. Log2 fold change values are also presented as heatmap; three biological replicates were performed. (B) H3K9Ac ChIP was performed on the Interferon-cured cells. The level of H3K9Ac for specific genes was quantified by qPCR, and values were normalized to those of interferon treated control cells. These levels were compared to HCV-infected cells and DAAs-cured cells. Log2 fold change values are also presented as heatmap; three biological replicates were performed. (PDF) [file pgen.1008181.s007.pdf]

Supplementary Figure 9

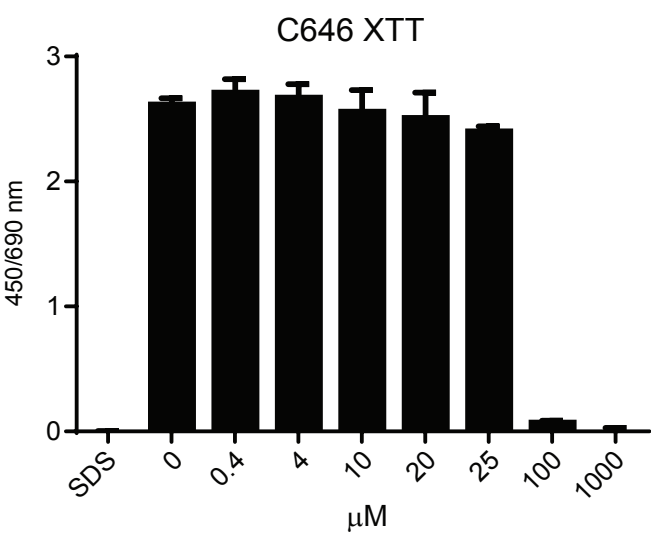

Supplement: S9 Fig — Huh7.5 cells were incubated with inhibitor in serial dilutions. The XTT assay was measured at 500 nm with reference of 690 nm. (PDF) [file pgen.1008181.s009.pdf]

Supplementary Figure 10

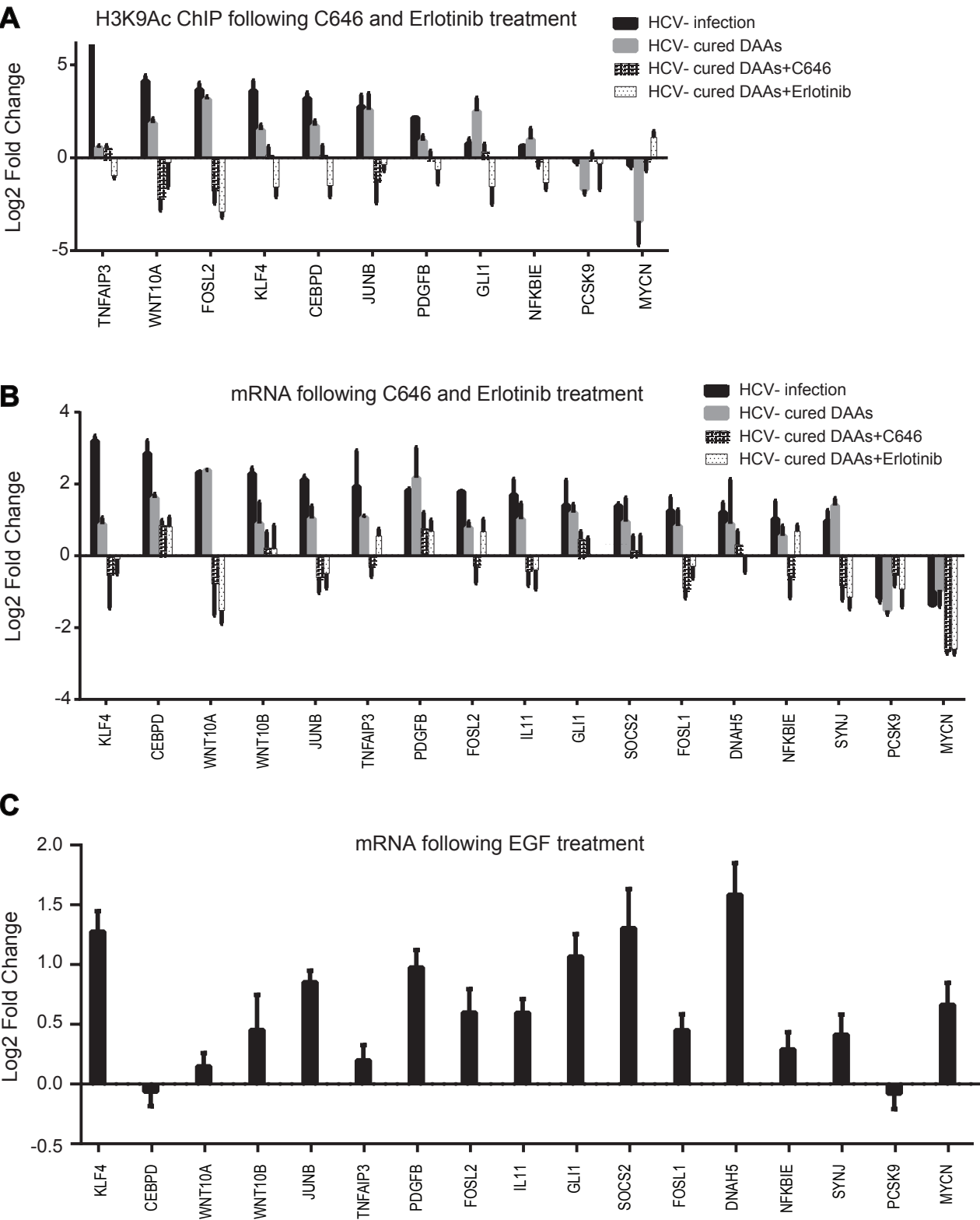

Supplement: S10 Fig — (A) Cured and control cells (non-infected Huh7.5 cells also treated with DAAs) were treated with 10 μM C646 or 1μM of EGFR inhibitor erlotinib for 1 week. Following treatment, H3K9Ac ChIP was performed on C646/erlotinib treated cured cells (normalized to control DAAs treated and then C646/erlotinib treated cells) compared to DAAs treated cells in the absence of C646/erlotinib (normalized to control DAAs treated cells), and to HCV infected cells (normalized to non-infected cells). The H3K9Ac level was quantified by qPCR with primers for specific genes. Values were normalized relative to qPCR for these genes following ChIP with normal Rabbit IgG Ab as control. The means ± SD of Log2 fold change values from three biological replicates are presented. (B) Cured and control cells were treated with 10 μM C646 or 1μM erlotinib for 1 week as described above. Following treatment, RNA was purified and qRT–PCR using primers for specific genes was performed. The means ± SD of Log2 fold change values from three biological replicates are presented for each gene. (C) Huh7.5 cell were treated with EGF for 48hr. Following treatment, RNA was purified and qRT–PCR using primers for specific genes was performed. The means ± SD of Log2 fold change values from three biological replicates are presented for each gene. (PDF) [file pgen.1008181.s010.pdf]
