# Supplementary material for: Hepatitis C virus leaves an epigenetic signature post cure of infection by direct-acting antivirals
Source: PLoS Genet. 2019 Jun 19;15(6):e1008181. doi: 10.1371/journal.pgen.1008181 (PMC6602261; doi:10.1371/journal.pgen.1008181)

Supplementary Figure 8

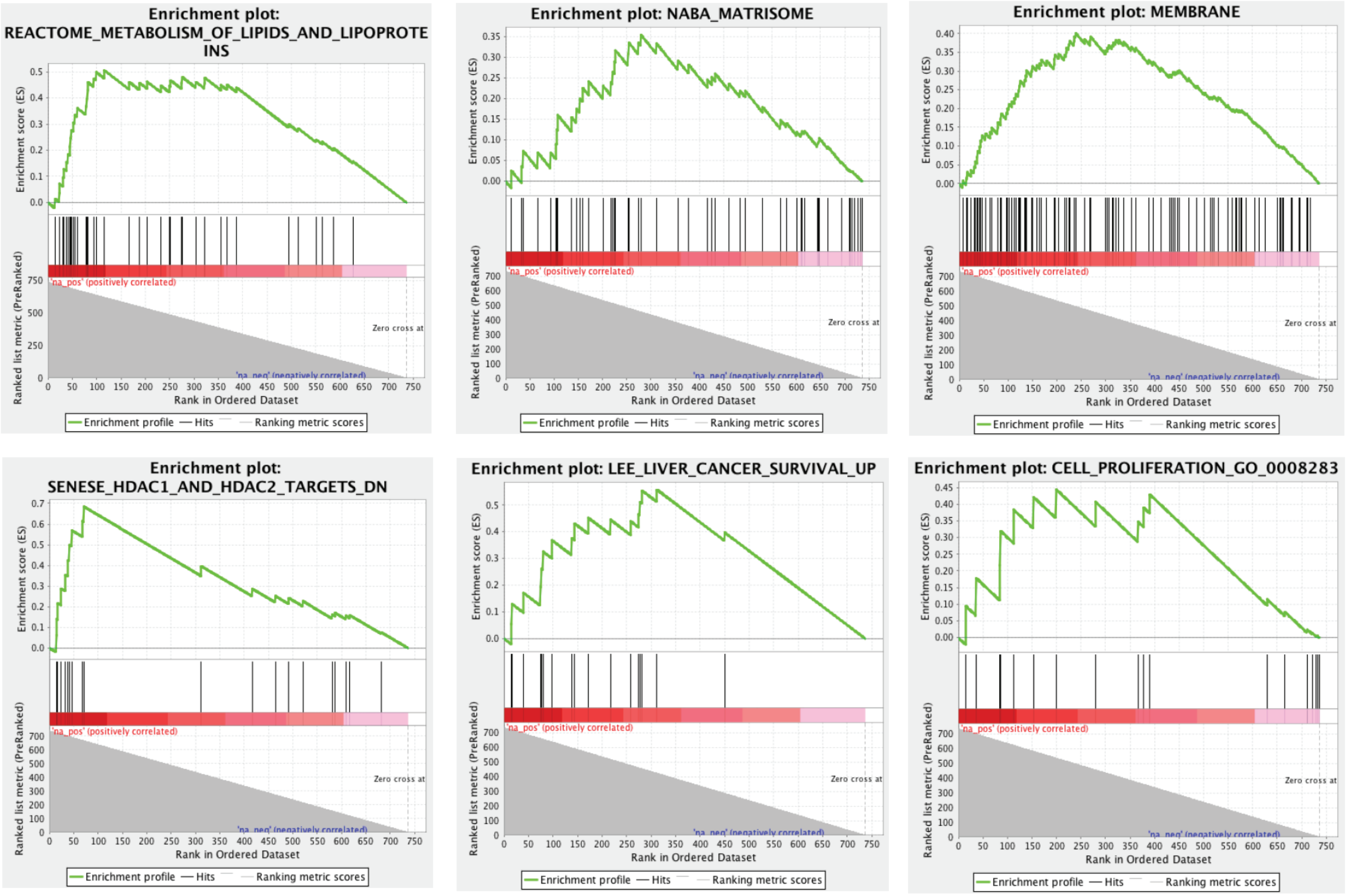

Supplement: S8 Fig — A ranked gene list was generated for the differential H3K9Ac ChIP-seq data according to the p value. This ranked list was used for Gene Set Enrichment Analysis (http://software.broadinstitute.org/gsea/index.jsp). Enrichment plots for significant gene sets are presented. (PDF) [file pgen.1008181.s008.pdf]
