# Supplementary material for: Hepatitis C virus leaves an epigenetic signature post cure of infection by direct-acting antivirals
Source: PLoS Genet. 2019 Jun 19;15(6):e1008181. doi: 10.1371/journal.pgen.1008181 (PMC6602261; doi:10.1371/journal.pgen.1008181)
Supplement: S1 Table — (PDF) [file pgen.1008181.s011.pdf]

Supplementary Table 1. Integration of changes in histone mark H3K4Me3 (ChIP-Seq) and mRNA expression (RNA-Seq) in HCV infected compared to non-infected cells

Gene Name	*Hg19 location*	*TSS_K4Me3 log2 fold change*	*TSS_K4Me3 adjusted P. value*	*mRNA log2 fold change*	*mRNA adjusted P. value*	*Gene Ontology**
**DDX60**	4:169239000-169240000	0.52	0.0458728	5.54	3.60E-92	Induce interferon and gene expression in response to viral infection.
**CEBPD**	8:48649000-48650000	0.41	0.0209813	3.32	2.70E-91	Cell differentiation, motility, growth arrest, proliferation, cell death, metabolism and immune responses
**WNT10A**	2:219762000-219763000	1.56	0.0002341	3.05	0.0038363	Activation of Wnt/β-catenin signaling
**KLF4**	9:110250000-110251000	1.68	4.56E-15	2.45	9.65E-107	High expression level is associated with vascular invasion and poor survival
**JUNB**	19:12902000-12903000	0.44	0.0027387	2.23	2.87E-272	Cell Growth/Signal Transduction Related.
**EDN1**	6:12291000-12292000	2.17	0.0068791	2.20	2.97E-159	Maintenance of vascular tone. Co-mitogenic activity, potentiating the effects of other growth factors such as PDGF.
**PDGFB**	22:39639000-39640000	0.57	1.67E-05	2.17	3.51E-72	Growth factor, regulation of embryonic development, cell proliferation, cell migration, survival and chemotaxis.
**FOSL1**	11:65666000-65667000	0.8	0.0120007	1.7	4.06E-13	Regulator of cell proliferation, differentiation, transformation, adhesion and migration.
**DUSP1**	5:172195000-172196000	1.28	0.00959	1.46	3.68E-224	Induced in oxidative/heat stress, negative regulation of cellular proliferation.
**APOA1-AS**	11:116707000-116708000	-0.6	0.0143811	-0.75	2.53E-20	The major protein component of high density lipoprotein (HDL) in plasma. The protein promotes cholesterol efflux from tissues to the liver for excretion.
**DLK1**	14:101194000-101195000	-1	0.0481319	-1.01	7.36E-65	Notch signaling pathway, regulation of gene expression, cell differentiation and development.
**PCSK9**	1:55506000-55507000	-0.6	1.21E-08	-1.21	8.23E-150	Liver development, regulation of receptor recycling and internalization, regulation of lipid metabolism, regulation of apoptosis.

*Gene Ontology-adjusted from GeneCards (https://www.genecards.org/)
