# Supplementary material for: Hepatitis C virus leaves an epigenetic signature post cure of infection by direct-acting antivirals
Source: PLoS Genet. 2019 Jun 19;15(6):e1008181. doi: 10.1371/journal.pgen.1008181 (PMC6602261; doi:10.1371/journal.pgen.1008181)
Supplement: S2 Table — (PDF) [file pgen.1008181.s012.pdf]

Supplementary Table 2. Integration of changes in histone mark H3K9Ac (ChIP-Seq) and mRNA expression (RNA-Seq) in HCV infected compared to non-infected cells for specific genes

Gene Name	*Hg19 location*	*TSS_K9Ac log2 fold change*	*TSS_K9Ac adjusted P. value*	*mRNA log2 fold change*	*mRNA adjusted P. value*	*Gene Ontology**
**CEBPD**	8:48649000-48650000	1.208	1.05E-08	3.32	2.70E-91	Cell differentiation, motility, growth arrest, proliferation, cell death, metabolism and immune responses
**PCSK9**	1:55506000-55507000	-1.12	2.41E-18	-1.21	8.23E-150	Liver development, regulation of receptor recycling and internalization, regulation of lipid metabolism, regulation of apoptosis
**FOSL2**	2:28613000-28614000	0.52	0.04595246	1.81	6.31E-225	Regulator of cell proliferation, differentiation, transformation, adhesion and migration
**KLF4**	9:110251000-110252000	1.03	2.93E-10	2.45	9.65E-107	High expression level is associated with vascular invasion and poor survival
**PDGFB**	22:39639000-39640000	0.94	2.30E-13	2.17	3.51E-72	Growth factor, regulation of embryonic development, cell proliferation, cell migration, survival and chemotaxis.
**WNT10A**	2:219761000-219762000	1.19	9.54E-06	3.05	0.0038363	Activation of Wnt/β-catenin signaling
**NFKBIE**	6:44231000-44232000	1.09	0.041	1.16	1.88E-37	Involved in inappropriate immune cell development and cell growth.
**GLI1**	12:57854000-57855000	1.13	5.81E-06	1.27	1.45E-07	Transcription factor, regulates stem cell proliferation, cell cycle progression and Wnt/β-catenin signaling.
**TNFAIP3**	6:138187000-138188000	0.8	2.54E-05	2.24	0	Involved in the cytokine-mediated immune and inflammatory responses.
**MYCN**	2:16080000-16081000	-0.78	2.25E-09	-1.83	1.51E-296	Transcription factor, regulates Notch Pathway, Apoptosis and Autophagy.

*Gene Ontology-adjusted from GeneCards (https://www.genecards.org/)
