# Supplementary material for: Hepatitis C virus leaves an epigenetic signature post cure of infection by direct-acting antivirals
Source: PLoS Genet. 2019 Jun 19;15(6):e1008181. doi: 10.1371/journal.pgen.1008181 (PMC6602261; doi:10.1371/journal.pgen.1008181)
Supplement: S4 Table — (PDF) [file pgen.1008181.s014.pdf]

Supplementary Table 4: Clinical and Pathological features of patients

Biopsy No.	Sample ID	Age	Sex	Cirrhosis yes/no	HCV infection/ SVR	Anti-HCV treatment	Time from treatment
1	RA-291	56	Male	Yes	HCV	NA	NA
2	1393542-9 Pre-treatment	57	Male	Yes	HCV	NA	NA
3	RA-367	74	Male	Yes	HCV	NA	NA
4	RA-374	58	Male	No	HCV	NA	NA
5	RA-390	57	Male	Yes	HCV	NA	NA
6	A-41	66	Female	No	HCV	NA	NA
7	A-51	59	Female	No	HCV	NA	NA
8	RA-312	57	Male	No	SVR	Viekirax+Exviera +Ribavirin 24 weeks	15 months
9	RA-323	64	Male	Yes	SVR	Viekirax+Exviera +Ribavirin 12 weeks	24 months
10	A-42	68	Female	No	SVR	Daclatasvir+ Asunaprevir +BMS-791325	12 months
11	A-52	60	Female	No	SVR	Daclatasvir+ Asunaprevir	12 months
12	RA-171	69	Male	Yes	NA	NA	NA
13	RA-235	68	Male	Yes	NA	NA	NA
14	RA-359	70	Female	Yes	NA	NA	NA
15	RA-244	75	Female	Yes	NA	NA	NA
16	RA-301	68	Female	No	NA	NA	NA
17	RA-307	72	Male	Yes	NA	NA	NA
